# Embedded FMCW Radar Target Detection and Tracking Based on Inter-Frame Differencing and Boundary-Adaptive CA-CFAR

**DOI:** 10.3390/s26165103

**Published:** 2026-08-12

**Authors:** Xun Zou, Wenyuan Feng, Bo Gao, Ni Gao, Jianzhong Chen

**Affiliations:** 1 Guangzhou Institute of Technology, Xidian University, Guangzhou 510555, China; 2 School of Electronic Engineering, Xidian University, Xi’an 710126, China; 3 College of Information and Control Engineering, Xi’an University of Architecture and Technology, Xi’an 710055, China; 4 Research and Education Development Institute, Xi’an Jiaotong University, Xi’an 710049, China; 5 Faculty of Electronic and Information Engineering, Xi’an Jiaotong University, Xi’an 710049, China

**Keywords:** FMCW radar, frame-differential range–Doppler enhancement, CA-CFAR, plot aggregation, finite-frame retained Kalman tracking, target re-association, firmware-path timing analysis

## Abstract

A compact 24 GHz FMCW radar board was evaluated for low-speed bicycle and small-vehicle sensing under strict memory and latency constraints. The hardware uses only 30 MHz modulation bandwidth, giving a nominal range resolution of about 5.0 m and a Doppler-bin spacing of about 2.57 m/s. Its small, incompletely calibrated antenna path also prevents any claim of high-angular-resolution imaging-radar performance. Within this constrained platform, the measured sequences reveal four coupled failure modes: static reflectors remain prominent in the range–Doppler map, useful low-Doppler responses are easily lost near the processed spectral boundary, weak plots do not always initiate a track, and short echo gaps can break otherwise continuous trajectories. To address these limitations, we combine frame-differential range–Doppler enhancement, quadrant-aware boundary-adaptive CA-CFAR, physically gated seed-growing initiation, and finite-frame retained Kalman tracking with SNR-weighted updates. In addition to natural bicycle and small-vehicle measurements, a labeled synthetic 64 by 32 range–Doppler benchmark is used to report Precision, Recall, F1-score, ROC/AUC, detection probability, and false alarms per frame for multiple CFAR variants. Public-radar tracking metrics are also reported on RadarScenes, a public RADIATE foggy sample, and nuScenes mini radar-only sequences with a bounded-approximation JPDA baseline. These public-radar results evaluate tracker-lifecycle and data-association behavior under public target-center observations; they are not presented as full validation of the board-specific RD-to-track pipeline. The evidence supports a bounded embedded-processing claim for this low-resolution board, not general applicability to high-resolution imaging radar systems.

## 1. Introduction

### 1.1. Research Background

Frequency-modulated continuous-wave (FMCW) radar is attractive for short-range transportation sensing because it can measure range and radial velocity under weak illumination, partial occlusion, and visually complex backgrounds [1,2]. Recent automotive radar research has expanded this role from simple ranging toward richer perception [3]. That expansion is important, but it also raises the demand for memory, computation, and deterministic scheduling on embedded platforms.

The present work targets a compact STM32F405-based 24 GHz FMCW board rather than a high-bandwidth imaging radar. Under this hardware constraint, the main problem is not only whether a target appears in one frame. The full processing chain must suppress static reflectors, preserve weak low-Doppler target energy near a processed spectral boundary, form stable plots from sparse detections, and maintain track identity through short echo gaps. Because these failure modes are coupled in the measured data, isolated module tuning is not enough.

### 1.2. Related Work

Classical radar detection is commonly built on range-Doppler processing followed by CFAR thresholding. Rohling introduced the CFAR framework for cluttered radar scenes, and Gandhi and Kassam analyzed why local-average CFAR can fail in nonhomogeneous backgrounds [4,5]. These methods remain attractive for embedded systems because they are deterministic and inexpensive. Their weakness is that clutter transitions, strong reflectors, and spectral boundaries can bias the training-cell estimate.

Recent radar perception research has expanded toward high-dimensional radar cubes, point clouds, temporal fusion, and multi-modal learning. Examples include real-time FMCW baseband processing [6], radar deep-learning surveys [7], range-azimuth-Doppler cube detection [8], multiframe 4D radar processing [9], RadarPillars [10], RaTrack [11], DoppDrive [12], and radar-LiDAR or radar-camera fusion methods [13,14,15]. Public datasets such as nuScenes, RadarScenes, and RADIATE have also strengthened evaluation for road-scene and adverse-weather radar perception [16,17,18]. These studies motivate temporal modeling and robust validation, but many assume richer sensors or larger processors than the compact board studied here.

Tracking and plot aggregation form the other part of the problem. Kalman filtering, gating, and data association are standard tools for radar tracking [19,20]. JPDA and JIPDA address measurement-origin uncertainty in multi-target scenes, but their full probabilistic forms can be costly on small microcontrollers [21,22]. DBSCAN is also widely used for point clustering, yet its radius and density thresholds are sensitive to sparse radar plots [23]. This paper, therefore, asks a narrower embedded question: under the memory, latency, and deterministic-control limits of the present board, how far can a fixed-size FMCW processing chain be improved by coupling frame-differential RD enhancement, boundary-adaptive CA-CFAR, physically gated seed-growing initiation, and finite-frame retained Kalman tracking?

### 1.3. Contributions

The main technical contributions are organized around the failure points observed in the measured radar frames:

1. The range–Doppler map is enhanced by differencing adjacent frames after the two-dimensional FFT, which suppresses stationary reflectors while avoiding the low-Doppler attenuation introduced by intra-frame slow-time differencing.
2. The CA-CFAR search region is restricted to the useful quadrant and compensated by a boundary-adaptive extension operator, so cells close to the processed spectral edge still use a complete training-window structure.
3. Target initiation is performed by seed-growing in physical plot variables. Spatial distance, radial-velocity consistency, point count, and accumulated SNR remain separate gates, which avoids a global neighborhood search and keeps every threshold interpretable.
4. Track continuity is improved by retaining Kalman-predicted states for short missed-detection intervals and by using SNR-weighted measurement updates when associated plots reappear.

The novelty is, therefore, system-level and implementation-oriented rather than a replacement of classical radar theory or a claim of superiority over every mature association framework. Classical MTI and clutter-map subtraction mainly modify temporal background suppression, edge-handled CFAR variants mainly modify the detection threshold, DBSCAN modifies plot aggregation, and JPDA/JIPDA modify association probability. The proposed method instead couples these stages under one fixed-size embedded data path, with explicit quadrant-conditioned boundary handling and physically separated seed-growth gates. Its claimed advantage is reproducible behavior on the present compact board, not a universal accuracy gain over higher-capacity radar pipelines.

To avoid terminology drift, the remainder of the manuscript uses four fixed names: frame-differential RD enhancement for the adjacent-frame spectral subtraction stage, boundary-adaptive CA-CFAR for the proposed edge-compensated detector, seed-growing initiation for the physically gated sparse-plot grouping stage, and finite-frame retained Kalman tracking for the bounded missed-frame lifecycle rule. Baseline names are kept only when they denote a distinct comparator, such as intra-frame differencing, zero padding, OS-CFAR, DBSCAN, or immediate deletion.

The experimental comparison is organized accordingly. For detection, the proposed chain is compared with intra-frame differencing and with multiple boundary-handling CFAR baselines under a shared mask and $P_{\mathrm{fa}}$. For plot aggregation, seed-growing is compared directly with DBSCAN on the same simulated *x*-*y*-$v_{r}$ point clouds. For tracking, finite-frame retained Kalman tracking is compared with the traditional immediate-deletion lifecycle on both natural radar measurements and controlled missed-detection conditions.

## 2. Principles of FMCW Radar Detection and Tracking

This section gives the theoretical baseline used by the proposed method. It intentionally describes the standard FMCW processing chain, conventional CA-CFAR decision rule, and classical tracking equations. The embedded improvements, including frame-differential RD enhancement, boundary-adaptive CA-CFAR, seed-growing initiation, and finite-frame retained Kalman tracking, are introduced separately in Section 3.

### 2.1. FMCW Signal Model and Range–Doppler Representation

In an FMCW radar, the transmitted chirp has a linear frequency slope $S=B/T_{\mathrm{chirp}}$, where *B* is the modulation bandwidth and $T_{\mathrm{chirp}}$ is the chirp duration. For a point target at range *R* and radial velocity *v*, mixing the received echo with the transmitted signal produces a beat component whose fast-time frequency is dominated by range and whose slow-time phase evolution is dominated by Doppler. Under the narrowband and far-field approximations, the range and Doppler frequencies satisfy

(1) $$f_{b}\approx \frac{2SR}{c},\;f_{D}=\frac{2v}{\lambda },\;\lambda =\frac{c}{f_{c}},$$

where *c* is the speed of light, $f_{c}$ is the carrier frequency, and λ is the wavelength.

The range resolution and maximum unambiguous radial velocity are determined by

(2) $$\Delta R=\frac{c}{2B},\;v_{\max}=\frac{\lambda }{4T_{c}},$$

where $T_{c}$ is the chirp repetition interval. In the experimental system, each frame contains $N_{c}=32$ chirps, each chirp retains $N_{s}=64$ valid samples, the carrier frequency is 24 GHz, the bandwidth is 30 MHz, and $T_{c}=76\;\mu s$. These parameters define a compact 64×32 range–Doppler observation per frame. They also imply a nominal range resolution of ΔR=5.0m, a maximum unambiguous radial velocity of approximately 41.1m/s, and a Doppler-bin spacing of approximately 2.57m/s. Therefore, the reported position and velocity statistics in the experiments should be interpreted as plot-centroid and Kalman-track consistency measures after smoothing, not as raw sub-meter range-bin accuracy. This resolution limit is important when comparing the present embedded platform with higher-bandwidth automotive or imaging radar systems.

Let $x_{k}\left[n,m\right]$ denote the complex sample in frame *k*, with fast-time index *n* and chirp index *m*. The standard two-dimensional FFT produces the range–Doppler spectrum

(3) $$\begin{array}{cc} S_{k}\left[p,q\right] & =\sum_{m=0}^{N_{c}-1}\left(\sum_{n=0}^{N_{s}-1}x_{k}\left[n,m\right]e^{-j2\pi pn/N_{s}}\right)e^{-j2\pi qm/N_{c}}. \end{array}$$

This spectrum is the common representation used by both classical radar processing and the embedded algorithm developed later.

### 2.2. Classical CA-CFAR Detection

After range–Doppler processing, target detection is commonly formulated as adaptive thresholding on a cell under test (CUT). CA-CFAR estimates the local background power from training cells surrounding the CUT while excluding guard cells that may contain target sidelobe energy [4]. If $T_{p,q}$ is the training-cell set around (p,q), the local average estimate is

(4) $${\hat{\sigma }}_{p,q}=\frac{1}{|T_{p,q}|}\sum_{\left(u,v\right)\in T_{p,q}}P\left[u,v\right],$$

where P[u,v] denotes the range–Doppler power. For a desired false-alarm probability $P_{\mathrm{fa}}$, the CA-CFAR scale factor and detection decision are

(5) $$\begin{array}{cc} \alpha & =|T_{p,q}|\left(P_{\mathrm{fa}}^{-1/|T_{p,q}|}-1\right),\\ B[p,q] & =1\;\left(P\left[p,q\right]>\alpha {\hat{\sigma }}_{p,q}\right). \end{array}$$

The advantage of CA-CFAR is its low computational cost and deterministic sliding-window structure. Its weakness is also well known: when the training cells are not statistically homogeneous, or when the CUT is close to a spectral boundary, the background estimate can be biased [5]. Section 3.2 addresses this weakness for the reduced embedded range–Doppler quadrant.

### 2.3. Radar Plot Tracking Fundamentals

CFAR detections are converted into radar plots that contain geometric and kinematic measurements, commonly range, azimuth, radial velocity, and signal strength. A tracking filter predicts the target state and then associates incoming plots with predicted measurements. For a Cartesian state

(6) $$x_{k}={\left[\begin{array}{cccc} x_{k} & y_{k} & v_{x,k} & v_{y,k} \end{array}\right]}^{⊤},$$

a constant-velocity prediction can be written as

(7) $$x_{k|k-1}=F_{\mathrm{CV}}x_{k-1|k-1},\;F_{\mathrm{CV}}=\left[\begin{array}{cccc} 1 & 0 & \Delta t & 0\\ 0 & 1 & 0 & \Delta t\\ 0 & 0 & 1 & 0\\ 0 & 0 & 0 & 1 \end{array}\right].$$

The nonlinear radar observation maps the Cartesian state to polar measurement space:

(8) $$h\left(x\right)=\left[\begin{array}{c} \sqrt{x^{2}+y^{2}}\\ \mathrm{atan}2(x,y)\\ \frac{xv_{x}+yv_{y}}{\sqrt{x^{2}+y^{2}}} \end{array}\right].$$

The coordinate convention used here follows the tracking code: *y* is the forward radar boresight direction and *x* is the lateral direction. Azimuth is, therefore, measured from the forward axis toward the lateral axis, which gives θ=atan2(x,y). This differs from the common mathematical convention atan2(y,x), but it is consistent with the radar plot conversion and the field-of-view drawings used in the experiments. For a measurement $z_{k}$, the innovation and Mahalanobis gating distance are

(9) $$e_{k}=z_{k}-h\left(x_{k|k-1}\right),\;d_{k}^{2}=e_{k}^{⊤}S_{k}^{-1}e_{k}.$$

The Kalman update then corrects the predicted state using the associated measurement [19]. In multi-target scenes, gating, association, target initiation, and track deletion determine whether detections become stable trajectories [20]. JPDA and JIPDA provide probabilistic association frameworks [21,22], but their full forms are costly for small microcontrollers. The next section, therefore, builds an embedded deterministic alternative on top of these standard principles.

## 3. Proposed Embedded Radar Target Detection and Tracking Algorithm

Section 2 gives the standard signal-processing and tracking foundation. This section focuses on the algorithmic modifications introduced for the embedded bicycle/vehicle radar experiment. The design goal is not to replace the classical range–Doppler–CFAR–Kalman chain, but to reshape it so that weak moving targets can be detected and retained under limited memory, deterministic execution, and sparse plot observations. The proposed chain contains four coupled innovations: frame-differential range–Doppler (RD) enhancement, quadrant-aware boundary-adaptive CA-CFAR, velocity-consistent seed-growing initiation, and finite-frame retained Kalman tracking with SNR-weighted updates.

**Figure 1:**
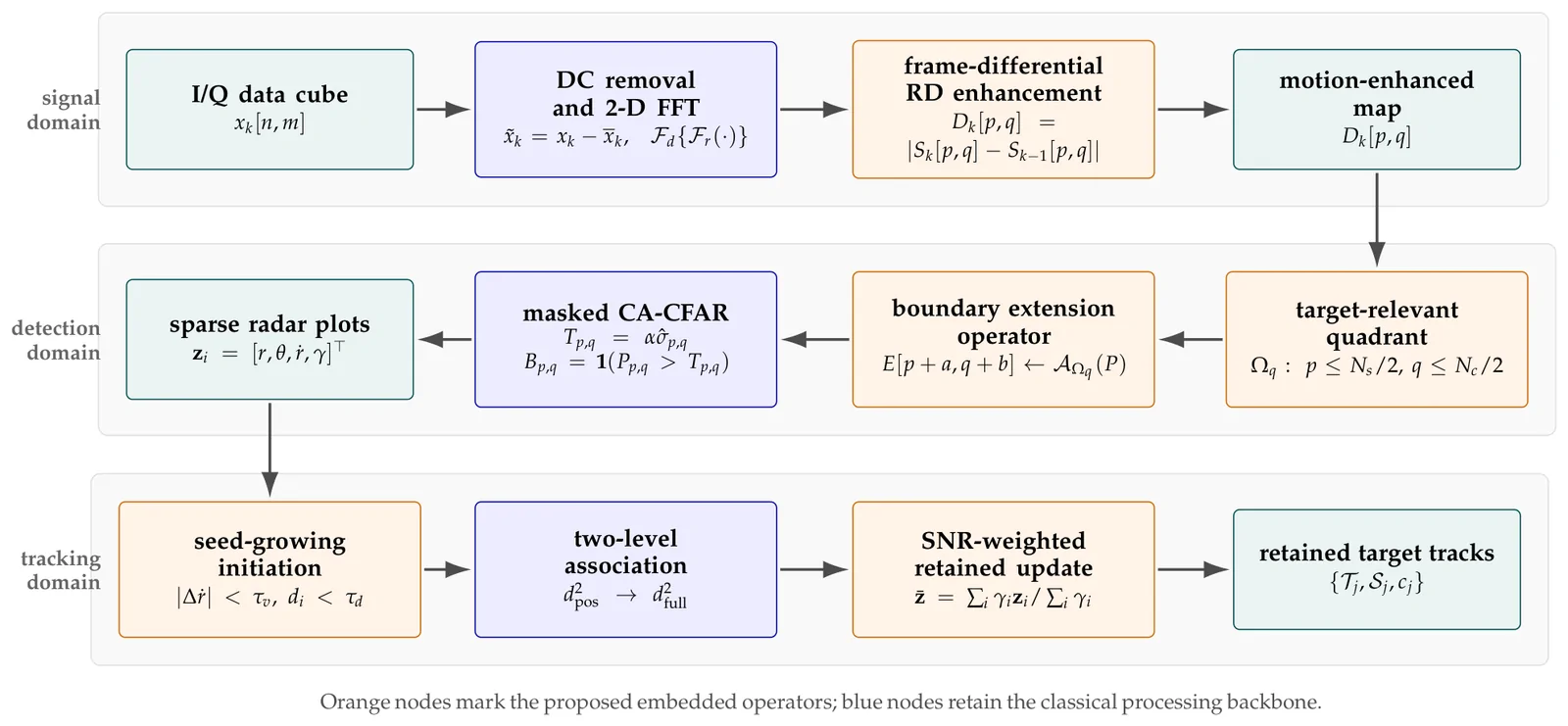
Operator-level schematic of the proposed embedded radar detection and tracking algorithm. Each node combines a processing action with the key mathematical operator used by the implementation, highlighting the four innovations of temporal RD enhancement, quadrant-conditioned CFAR extension, seed-growing initiation, and retained SNR-weighted tracking.

The operator-level structure is shown in Figure 1. Unlike a generic flowchart, the diagram exposes the representation change at each stage: raw I/Q samples are converted into a motion-enhanced range–Doppler map, the map is restricted and extended in a target-relevant quadrant before CFAR, and the resulting sparse plots are transformed into temporally retained tracks.

### 3.1. Frame-Differential Range–Doppler Enhancement

Let $x_{k}\left[n,m\right]$ denote the complex I/Q sample of frame *k*, where $n=0,\ldots ,N_{s}-1$ is the fast-time sample index and $m=0,\ldots ,N_{c}-1$ is the chirp index. In the implemented system, $N_{s}=64$ and $N_{c}=32$. Before the Fourier transforms, a frame-wise range DC component is removed to suppress the stationary leakage common to all range samples:

(10) $${\tilde{x}}_{k}\left[n,m\right]=x_{k}\left[n,m\right]-\frac{1}{N_{s}}\sum_{n^{\prime }=0}^{N_{s}-1}x_{k}\left[n^{\prime },m\right].$$

Hamming windows are then applied along both the range and Doppler dimensions. The range–Doppler spectrum is

(11) $$\begin{array}{cc} S_{k}\left[p,q\right] & ={\mathrm{fftshift}}_{q}\left\{\sum_{m=0}^{N_{c}-1}w_{d}\left[m\right]\;X_{k}\left[p,m\right]\;e^{-j2\pi qm/N_{c}}\right\},\\ X_{k}\left[p,m\right] & =\sum_{n=0}^{N_{s}-1}w_{r}\left[n\right]{\tilde{x}}_{k}\left[n,m\right]e^{-j2\pi pn/N_{s}}. \end{array}$$

The key enhancement step is performed after Doppler processing rather than inside a single frame. The enhanced RD-magnitude map is defined as

(12) $$M_{k}\left[p,q\right]=\left|S_{k}\left[p,q\right]-S_{k-1}\left[p,q\right]\right|,\;k\geq 2.$$

This differs from conventional intra-frame MTI filtering, which differentiates adjacent chirps before Doppler FFT. Intra-frame differencing introduces a deterministic high-pass response along slow time and may attenuate weak low-Doppler targets. Frame-differential RD enhancement in (12) instead compares complete RD spectra across adjacent radar frames. Therefore, static background and slowly varying clutter are cancelled at the spectral-image level, while a moving object remains because its range bin, Doppler bin, or scattering amplitude changes over time.

The velocity response can be seen from a simplified coherent point-target model. Suppose that a target remains in the same RD bin over two adjacent frames, has constant complex amplitude *A*, and only its carrier phase changes between frame starts separated by $T_{f}$. Then

(13) $$S_{k}\left[p_{0},q_{0}\right]=Ae^{j\phi_{k}},\;\phi_{k}-\phi_{k-1}=\frac{4\pi v_{r}T_{f}}{\lambda },$$

and the frame-differenced magnitude becomes

(14) $$\left|S_{k}\left[p_{0},q_{0}\right]-S_{k-1}\left[p_{0},q_{0}\right]\right|=2\left|A\right|\left|\sin\left(\frac{2\pi v_{r}T_{f}}{\lambda }\right)\right|.$$

Thus, perfectly coherent same-bin targets have velocity-dependent gain and possible nulls when $v_{r}=n\lambda /\left(2T_{f}\right)$, where *n* is an integer. In the present system $T_{f}=0.05\;s$ and λ≈12.5mm, so the first spacing between such idealized nulls is about 0.125m/s. Exact cancellation requires unchanged amplitude, unchanged range and Doppler bins, and stable scattering phase. In measured bicycle and vehicle frames, bin migration, acceleration, window leakage, and amplitude scintillation break these conditions. The operation should, therefore, be interpreted as a motion-sensitive background suppressor, not as a uniformly gain-preserving detector for all radial velocities. Very slow or exactly phase-periodic returns can be attenuated, which is why the following seed-growing stage rejects near-static plots explicitly.

For embedded execution, the detector is restricted to the physically useful quadrant

(15) $$\Omega_{q}=\left\{\left(p,q\right)|1\leq p\leq N_{s}/2,\;1\leq q\leq N_{c}/2\right\}.$$

This reduction is not a cosmetic cropping operation. It encodes the experimental observation that the useful bicycle and vehicle returns are concentrated in one range–velocity quadrant after the selected Doppler-axis convention. The CFAR search area is, therefore, reduced from $N_{s}N_{c}$ cells to $N_{s}N_{c}/4$ cells while preserving the target-relevant region. This is one of the reasons why the method is suitable for low-power embedded deployment.

The quadrant choice is a platform- and scenario-specific convention rather than a general radar assumption. The Doppler axis is FFT-shifted, and the first retained Doppler half corresponds to the selected approaching-target sign convention used in the data scripts. If a deployment contains both motion directions or a different Doppler sign convention, $\Omega_{q}$ must be mirrored or expanded before applying the same boundary-extension logic. Thus, the proposed detector should be understood as a quadrant-conditioned embedded implementation, not as a restriction inherent to FMCW radar.

### 3.2. Quadrant-Aware Boundary-Adaptive CA-CFAR

Conventional CA-CFAR assumes a complete training window around each cell under test (CUT). Near the edge of $\Omega_{q}$, this assumption is violated; either the boundary cells are skipped, or the reference set is shortened. Both choices are undesirable for weak low-speed targets because the threshold becomes unstable exactly where the useful Doppler support is narrow.

To avoid this problem, the implementation constructs an extended spectrum before CFAR. Let

(16) $$P\left[p,q\right]=M_{k}\left[p,q\right],\;\left(p,q\right)\in \Omega_{q}$$

be the quadrant RD magnitude map. For a CFAR mask with half sizes *a* and *b* along range and Doppler, respectively, the extended matrix *E* is first initialized by embedding the valid quadrant:

(17) $$E\left[p+a,q+b\right]=P\left[p,q\right],\;1\leq p\leq N_{r},\;1\leq q\leq N_{d}.$$

The out-of-domain cells are filled using a quadrant-aware anchoring rule:

(18) $$\begin{array}{cc} E[p+a,q+b] & =\left\{\begin{array}{cc} P[1,1], & p<1,\\ P[N_{r},1], & p>N_{r},\\ P[\mathrm{clip}\left(p,1,N_{r}\right),1],\; & q<1\;\mathrm{or}\;q>N_{d},\\ P[p,q], & \mathrm{otherwise}. \end{array}\right. \end{array}$$

Unlike zero padding, (18) does not artificially lower the reference power near the boundary. Unlike full mirror padding, it does not replicate high-energy target ridges symmetrically into invalid Doppler regions. Instead, the Doppler-side padding is anchored to the first valid Doppler column, which acts as a conservative background profile for the quadrant detector.

In implementation, the range boundary is filled by the first-column endpoint because the processed quadrant has no valid samples above or below the retained range interval. The Doppler boundary is filled row-by-row from the first valid Doppler column. This keeps the local range profile while avoiding symmetric replication into invalid Doppler bins. This boundary rule is heuristic and scenario-conditioned. It is appropriate for the retained quadrant and Doppler-sign convention used in the measured data, but it is not claimed to be CFAR-optimal for arbitrary clutter fields, bistatic geometries, or opposite Doppler-sign conventions.

The CA-CFAR window is represented by a binary mask W[Δp,Δq], where the guard region around the CUT is set to zero. If $N_{W}=\sum W$ is the number of training cells, the local noise estimate and adaptive threshold are

(19) $$\begin{array}{cc} {\hat{\sigma }}_{p,q} & =\frac{1}{N_{W}}\sum_{\Delta p=-a}^{a}\sum_{\Delta q=-b}^{b}W\left[\Delta p,\Delta q\right]\;E\left[p+a+\Delta p,q+b+\Delta q\right], \end{array}$$

(20) $$\begin{array}{cc} T_{p,q} & =\alpha {\hat{\sigma }}_{p,q},\;\alpha =N_{W}\left(P_{\mathrm{fa}}^{-1/N_{W}}-1\right). \end{array}$$

Because P[p,q] is the magnitude of a frame-differenced complex RD spectrum, the exponential power-sample assumption behind the classical CA-CFAR false-alarm formula is not strictly satisfied. In this implementation, $P_{\mathrm{fa}}$ is, therefore, used as a nominal threshold-control parameter that fixes the common scale factor for matched comparisons. The achieved false-alarm behavior is evaluated empirically in the labeled synthetic benchmark and the boundary sweep rather than inferred solely from (20). The detection decision is

(21) $$B_{k}\left[p,q\right]=1\;\left(P\left[p,q\right]>T_{p,q}\right),\;\left(p,q\right)\in \Omega_{q}.$$

This formulation explains the main novelty of the detector: the boundary condition is moved from the inner CFAR loop into a precomputed extension operator. The detector, therefore, preserves a uniform sliding-window computation while producing more stable thresholds near spectral edges.

### 3.3. Plot Formation and Seed-Growing Target Initiation

Each CFAR detection is converted into a radar plot

(22) $$z_{i}={\left[r_{i},\;\theta_{i},\;{\dot{r}}_{i},\;\gamma_{i}\right]}^{⊤},$$

where $r_{i}$ is range, $\theta_{i}$ is azimuth, ${\dot{r}}_{i}$ is radial velocity, and $\gamma_{i}$ is SNR. For 3D measurement, the elevation angle can be inserted before the velocity term.

The tracker receives a sparse plot set, not a dense point cloud. Therefore, a global density clustering method is unnecessary and may be unstable when the number of detections is small. The implemented target initiation uses a deterministic seed-growing operator. Let $U_{k}$ be the set of unassociated plots in frame *k*. Static or near-zero-velocity plots are rejected before allocation:

(23) $$U_{k}^{\mathrm{dyn}}=\left\{z_{i}\in U_{k}|\left|{\dot{r}}_{i}\right|>v_{\mathrm{static}}\right\}.$$

Starting from a seed $z_{s}$, a candidate cluster C is expanded by scanning the remaining dynamic plots. Since Doppler measurements are ambiguous modulo the maximum unambiguous radial velocity, a velocity unwrapping operator is used:

(24) $$\begin{array}{cc} \mathrm{unwrap}({\dot{r}}_{i};\mu_{\dot{r}}) & ={\dot{r}}_{i}+2v_{\max}\ell^{★},\\ \ell^{★} & =\underset{\ell \in Z}{arg min}\left|{\dot{r}}_{i}+2\ell v_{\max}-\mu_{\dot{r}}\right|. \end{array}$$

Here, $v_{\max}$ is the maximum unambiguous radial speed defined by the chirp repetition interval, so the measured Doppler velocity is periodic with period $2v_{\max}$. The implementation selects the alias that is closest to the current cluster or track velocity expectation $\mu_{\dot{r}}$. This operation handles bin-to-bin Doppler wrapping inside the fixed 32-chirp frame, but it does not remove the need to choose the correct physical Doppler sign convention. In the firmware path, the velocity axis is generated from $-v_{\max}$ to $+v_{\max}$, and the detector then operates on the retained quadrant used by the measured bicycle and vehicle sequences. If the radar mounting direction, target approach direction, or sign convention changes, the retained Doppler region and the boundary-sensitive ROI must be mirrored or expanded before applying the same CFAR and seed-growing rules.

A plot is absorbed into the seed cluster only when both velocity consistency and polar-plane proximity are satisfied:

(25) $$\begin{array}{cc} \left|\mathrm{unwrap}\left({\dot{r}}_{i};\mu_{\dot{r}}\right)-\mu_{\dot{r}}\right| & <\tau_{v}, \end{array}$$

(26) $$\begin{array}{cc} d_{i}\left(z_{i},\mu_{C}\right) & =\sqrt{r_{i}^{2}+\mu_{r}^{2}-2r_{i}\mu_{r}\cos\left(\theta_{i}-\mu_{\theta }\right)}<\tau_{d}. \end{array}$$

The cluster center, point count, and accumulated SNR are updated online:

(27) $$\mu_{C}=\frac{1}{\left|C\right|}\sum_{z_{i}\in C}z_{i},\;\Gamma_{C}=\sum_{z_{i}\in C}\gamma_{i}.$$

The cluster is promoted to a new track only when the following multi-condition validity test is satisfied:

(28) $$\begin{array}{cc} I(C) & =1\;\left[\left|C\right|\geq N_{\min}\right]1\;\left[|\mu_{\dot{r}}|>v_{\min}\right]\\ \; & \;\times 1\;\left[\Gamma_{C}>\Gamma_{\min}^{*}\right]1\;\left[d_{\mathrm{adj}}\left(C\right)>d_{\min}\right]. \end{array}$$

The SNR threshold is selected according to occlusion status:

(29) $$\Gamma_{\min}^{*}=\left\{\begin{array}{cc} \Gamma_{\mathrm{obs}}, & \mathrm{partially}\;\mathrm{occluded}\;\mathrm{or}\;\mathrm{behind}\;\mathrm{an}\;\mathrm{active}\;\mathrm{target},\\ \Gamma_{\mathrm{normal}}, & \mathrm{normal}\;\mathrm{case}. \end{array}\right.$$

**Figure 2:**
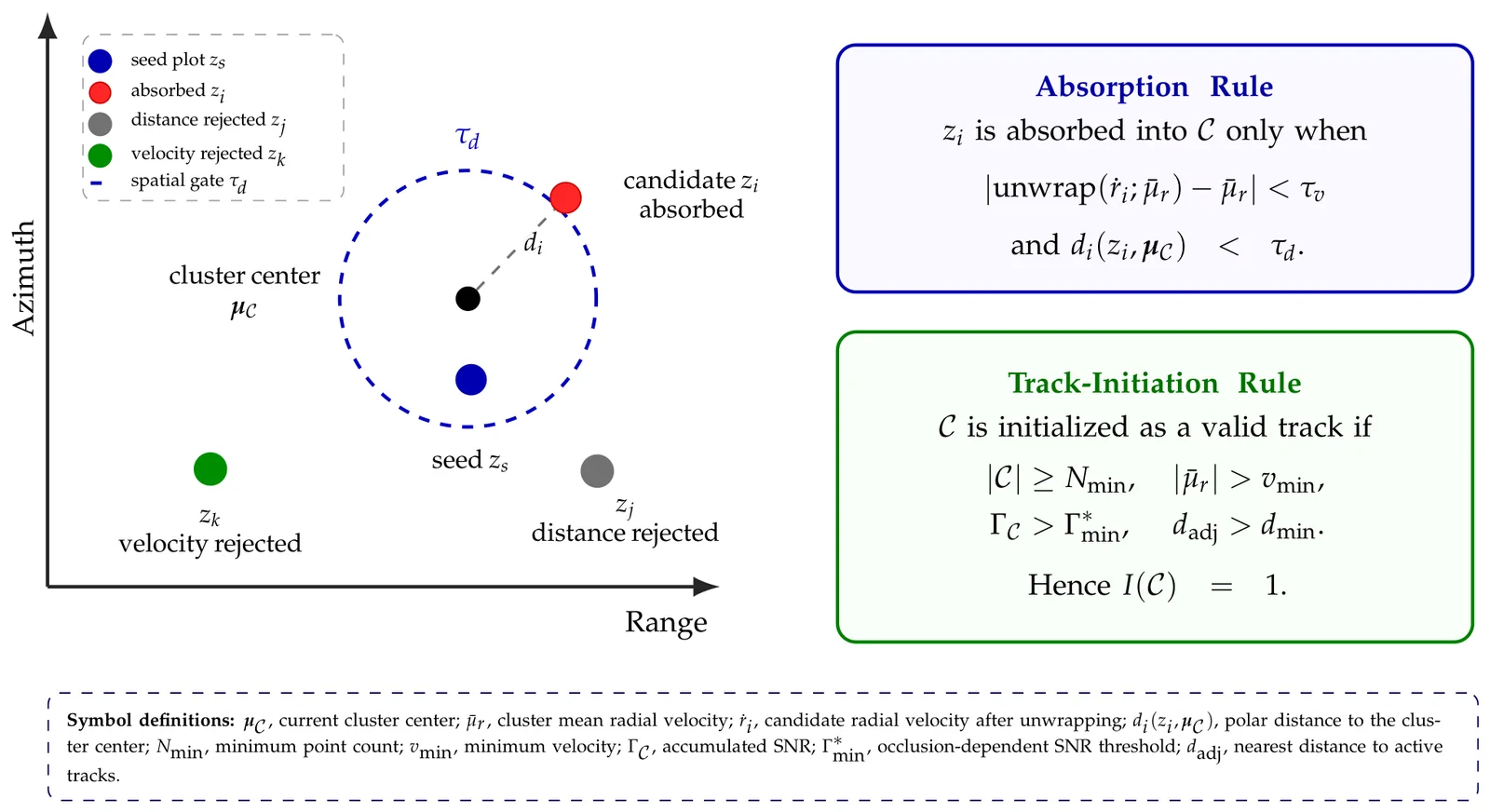
Geometry of the seed-growing target-initiation rule. A candidate plot must satisfy both the radial-velocity gate and the spatial gate with respect to the current aggregation center. The final track-initiation decision further depends on point count, accumulated SNR, velocity magnitude, occlusion status, and adjacency to existing tracks.

Here $d_{\mathrm{adj}}\left(C\right)$ is the minimum Cartesian distance between the new candidate center and existing active tracks:

(30) $$d_{\mathrm{adj}}\left(C\right)=\left\{\begin{array}{cc} \min_{T_{j}\in A_{k}}{∥\left[\begin{array}{c} \mu_{r}\sin\mu_{\theta }\\ \mu_{r}\cos\mu_{\theta } \end{array}\right]-\left[\begin{array}{c} {\hat{x}}_{j,k|k-1}\\ {\hat{y}}_{j,k|k-1} \end{array}\right]∥}_{2},\; & |A_{k}|>0,\\ +\infty , & |A_{k}|=0, \end{array}\right.$$

where $A_{k}$ is the set of active tracks. A candidate is treated as partially occluded or behind an active target when it lies in a similar bearing interval but at a larger range than that track. This adjacency check suppresses false initiation caused by sidelobes or fragmented detections around an already tracked target. The seed-growing geometry is illustrated in Figure 2.

### 3.4. Gating Association with SNR-Weighted Measurement Update

For each active track, the predicted state is transformed into measurement space through the nonlinear radar observation model:

(31) $$h\left(x\right)=\left[\begin{array}{c} \sqrt{x^{2}+y^{2}}\\ \mathrm{atan}2(x,y)\\ \frac{xv_{x}+yv_{y}}{\sqrt{x^{2}+y^{2}}} \end{array}\right].$$

where the three components correspond to range, azimuth, and radial velocity. The association stage uses a two-level gate. First, a coarse Mahalanobis test is computed while suppressing the Doppler residual, which prevents velocity ambiguity from rejecting a geometrically plausible plot too early:

(32) $$d_{\mathrm{pos}}^{2}={\tilde{e}}^{⊤}C^{-1}\tilde{e},\;\tilde{e}={\left[\begin{array}{ccc} e_{r} & e_{\theta } & 0 \end{array}\right]}^{⊤}.$$

If the plot passes this outer gate, the full residual

(33) $$d_{\mathrm{full}}^{2}=e^{⊤}C^{-1}e,\;e=z-h\left({\hat{x}}_{k|k-1}\right),$$

is used for final scoring. The score used in competition between tracks is

(34) $$J\left(z,T\right)=\log|C|+d_{\mathrm{full}}^{2}.$$

This makes the association deterministic: each plot is assigned to the track with the smallest gated score, and tracks with nonzero radial velocity are given priority over static or ghost-labeled hypotheses during association.

When multiple plots are associated with one track, the measurement used for Kalman update is not a simple arithmetic center. The implementation forms an SNR-weighted centroid:

(35) $${\bar{z}}_{k}=\sum_{i\in M_{k}}\omega_{i}z_{i},\;\omega_{i}=\frac{\gamma_{i}}{\sum_{j\in M_{k}}\gamma_{j}},$$

where $M_{k}$ is the set of associated plots. This update makes high-SNR returns contribute more strongly to the track correction while still using weaker neighboring points to estimate the target extent.

### 3.5. Retained Kalman Prediction and Track Lifecycle Control

The implemented 2D tracker uses a constant-acceleration state vector

(36) $$x_{k}={\left[\begin{array}{cccccc} x_{k} & y_{k} & v_{x,k} & v_{y,k} & a_{x,k} & a_{y,k} \end{array}\right]}^{⊤}.$$

The prediction model is

(37) $$x_{k|k-1}=Fx_{k-1|k-1},$$

with

(38) $$F=\left[\begin{array}{cccccc} 1 & 0 & \Delta t & 0 & \frac{1}{2}\Delta t^{2} & 0\\ 0 & 1 & 0 & \Delta t & 0 & \frac{1}{2}\Delta t^{2}\\ 0 & 0 & 1 & 0 & \Delta t & 0\\ 0 & 0 & 0 & 1 & 0 & \Delta t\\ 0 & 0 & 0 & 0 & 1 & 0\\ 0 & 0 & 0 & 0 & 0 & 1 \end{array}\right].$$

The covariance prediction and Kalman update follow

(39) $$\begin{array}{cc} P_{k|k-1} & =FP_{k-1|k-1}F^{⊤}+Q, \end{array}$$

(40) $$\begin{array}{cc} S_{k} & =H_{k}P_{k|k-1}H_{k}^{⊤}+R, \end{array}$$

(41) $$\begin{array}{cc} K_{k} & =P_{k|k-1}H_{k}^{⊤}S_{k}^{-1}, \end{array}$$

(42) $$\begin{array}{cc} x_{k|k} & =x_{k|k-1}+K_{k}\left({\bar{z}}_{k}-h\left(x_{k|k-1}\right)\right). \end{array}$$

Here $H_{k}$ is the EKF Jacobian of the nonlinear measurement function h(x), evaluated at ${\hat{x}}_{k|k-1}$. With $\rho =\sqrt{x^{2}+y^{2}}$, $s=xv_{x}+yv_{y}$, and a small lower bound on ρ in implementation to avoid division by zero, the Jacobian is

(43) $$H_{k}={\left[\begin{array}{cccccc} x/\rho & y/\rho & 0 & 0 & 0 & 0\\ y/\rho^{2} & -x/\rho^{2} & 0 & 0 & 0 & 0\\ v_{x}/\rho -sx/\rho^{3} & v_{y}/\rho -sy/\rho^{3} & x/\rho & y/\rho & 0 & 0 \end{array}\right]}_{x={\hat{x}}_{k|k-1}}.$$

This is an EKF-style local linearization, not a globally linear measurement model.

For the 2D constant-acceleration mode used in the experiments, Δt=0.05s, and the state order is ${\left[x,y,v_{x},v_{y},a_{x},a_{y}\right]}^{T}$. The process covariance uses the standard constant-acceleration block form in (38) and is scaled per Cartesian dimension by $\left(0.5\right|a_{\max}{\left|\right)}^{2}$. The sign stored in the project configuration only indicates the limiting acceleration direction used by the legacy firmware parameter file; the covariance itself uses the nonnegative magnitude $|a_{\max}|$. With $|a_{\max}|=0.1\;m/s^{2}$, this gives a per-dimension process-variance scale of $2.5\times 10^{-3}$. The initial covariance, measurement spread, centroid covariance, and Doppler-unrolling constants are reported in the experiment configuration rather than as a separate method table.

The tracker does not immediately delete an active track when no measurement is associated. Instead, the predicted state is retained and the miss counter is increased:

(44) $$c_{k}=\left\{\begin{array}{cc} 0, & \mathrm{if}\;\mathrm{associated},\\ c_{k-1}+1, & \mathrm{if}\;\mathrm{missed}. \end{array}\right.$$

The state transition rule is

(45) $$S_{k}=\left\{\begin{array}{cc} \mathrm{ACTIVE}, & S_{k-1}=\mathrm{DETECTION}\;\mathrm{and}\;c_{\mathrm{hit}}>T_{\mathrm{act}},\\ \mathrm{FREE}, & S_{k-1}=\mathrm{DETECTION}\;\mathrm{and}\;c_{\mathrm{miss}}>T_{\det},\\ \mathrm{FREE}, & S_{k-1}=\mathrm{ACTIVE}\;\mathrm{and}\;c_{\mathrm{miss}}>T_{\mathrm{free}},\\ S_{k-1}, & \mathrm{otherwise}. \end{array}\right.$$

This finite-frame retained Kalman mechanism is a practical compromise between aggressive deletion and indefinite track persistence. It allows short-term missed detections, shadowing, or temporary CFAR failure to be bridged by prediction, while still bounding memory and preventing stale tracks from accumulating.

Overall, the novelty of the proposed embedded algorithm lies in the coupling between signal-domain enhancement and tracker-domain reliability. Frame-differential RD enhancement produces a motion-sensitive RD map; boundary-adaptive CA-CFAR stabilizes threshold estimation in the reduced quadrant; seed-growing initiation converts sparse detections into reliable target candidates without density clustering and finite-frame retained Kalman tracking preserves target identity through short detection gaps. These mechanisms are deliberately lightweight, but each one addresses a concrete failure mode observed in embedded radar experiments.

## 4. Experimental Analysis

### 4.1. Experimental Platform and Parameter Settings

**Figure 3:**
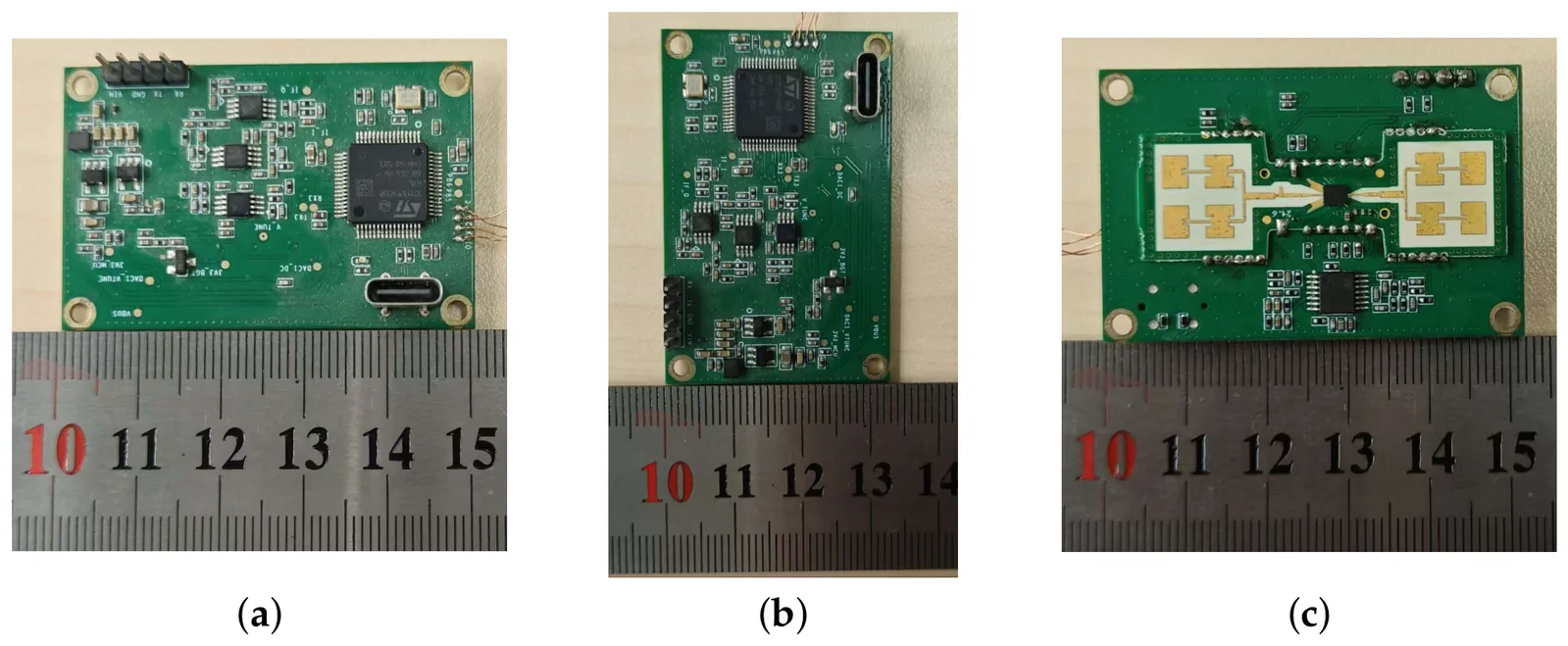
Photographs of the embedded FMCW radar board used in the experiments. The controller side includes the STM32F405 microcontroller, USB Type-C connector, UART/power header, and analog test nodes, while the opposite side exposes the 24 GHz planar antenna structures and RF front-end layout. (**a**) Controller and interface side; (**b**) MCU-side rotated view; (**c**) Antenna and RF side.

The experimental platform was built on a compact self-developed 24 GHz FMCW radar printed circuit board from Xi’an, China, as shown in Figure 3. The controller side integrates an STM32F405RGT6 microcontroller, a USB Type-C interface, UART pins marked RX/TX/GND/VIN, power-regulation circuits, and several analog/radar test nodes such as $V_{\mathrm{TUNE}}$, DAC/DC, MCU 3.3 V, and VBUS. The STM32F405RGT6 belongs to the ARM Cortex-M4F class and provides a maximum core frequency of 168 MHz, 1 MB on-chip Flash, 192 KB SRAM, and single-precision floating-point support. These device-level resources are modest compared with application processors typically used for high-dimensional radar perception, which is why the present processing chain uses fixed-size buffers, deterministic control flow, and bounded plot/track capacities. The antenna side contains the planar 24 GHz transmit/receive antenna structures and the corresponding RF front-end routing. The ruler in the photographs indicates that the board is approximately in the 5 cm class, which is consistent with the intended low-power embedded deployment rather than a laboratory rack-mounted radar setup.

During data acquisition, the radar board produced raw I/Q frames that were transferred to the host for algorithm evaluation and then processed using the same fixed-size structure intended for MCU deployment. This host-side evaluation preserves the frame format and fixed-capacity data path, but it should not be read as direct on-board timing or memory validation. Each frame contains $N_{c}=32$ chirps, and each chirp retains $N_{s}=64$ valid fast-time samples. The frame rate is 20 Hz, corresponding to a 50 ms frame interval. With a 24 GHz carrier, 30 MHz modulation bandwidth, and 1 MHz sampling rate, the system provides a short-range sensing configuration suitable for bicycle and small-vehicle experiments. The processing chain uses a 64-point range FFT and a 32-point Doppler FFT, followed by quadrant-restricted frame-differential RD enhancement, boundary-adaptive CA-CFAR, seed-growing initiation, and finite-frame retained Kalman tracking.

The available experimental board provides low-dimensional radar plots rather than a high-resolution 4D radar cube. The recorded plot fields are range, Doppler or radial velocity, azimuth, elevation, and SNR. These variables are sufficient for the board-level detection and tracking comparisons reported below. They are not sufficient for a calibrated angular-accuracy study, because the current project materials do not include the full antenna geometry, the angle-estimation implementation, or an independent angular calibration. The tracking results are, therefore, reported as system-level plot-tracking results for this board, and the conclusions are not generalized to high-angular-resolution MIMO radar platforms.

The tested data include a low-speed bicycle sequence, a small-vehicle sequence, and cluttered background measurements. The main platform and default board-level processing parameters are summarized in Table 1. The table separates physical radar parameters, frame structure, and representative algorithm settings. Experiment-specific tracking parameters are stated again in the corresponding evaluation subsection because the natural-data tracker, public-data tracker, and synthetic detection benchmark use different observation scales.

### 4.2. Evaluation Metrics and Labeling Protocol

The boundary indicators used below are defined as follows. Let $\Omega_{\mathrm{ROI}}$ denote the manually selected boundary-sensitive ROI, which covers all range rows in the last five Doppler columns of the retained range–Doppler quadrant. Let $B_{k}\left(r,d\right)\in \left\{0,1\right\}$ be the binary detection map for frame *k*, $P_{k,\mathrm{dB}}\left(r,d\right)$ the RD magnitude in dB, and $T_{k}\left(r,d\right)$ the CFAR threshold. The ROI detection count is

(46) $$N_{\mathrm{ROI}}\left(k\right)=\sum_{\left(r,d\right)\in \Omega_{\mathrm{ROI}}}B_{k}\left(r,d\right).$$

The ROI contrast and boundary-threshold coefficient of variation are

(47) $$\begin{array}{cc} C_{\mathrm{ROI}}\left(k\right) & =\frac{1}{|\Omega_{\mathrm{ROI}}|}\sum_{\left(r,d\right)\in \Omega_{\mathrm{ROI}}}P_{k,\mathrm{dB}}\left(r,d\right)-\frac{1}{|{\bar{\Omega }}_{\mathrm{ROI}}|}\sum_{\left(r,d\right)\in {\bar{\Omega }}_{\mathrm{ROI}}}P_{k,\mathrm{dB}}\left(r,d\right),\\ {\mathrm{CV}}_{\mathrm{thr}}\left(k\right) & =\frac{\mathrm{std}\{T_{k}\left(r,d\right):\left(r,d\right)\in \Omega_{\mathrm{ROI}}\}}{\mathrm{mean}\{T_{k}\left(r,d\right):\left(r,d\right)\in \Omega_{\mathrm{ROI}}\}}. \end{array}$$

Here, ${\bar{\Omega }}_{\mathrm{ROI}}$ is the complement of the selected ROI within the retained quadrant. These quantities measure boundary sensitivity and threshold stability for selected frames. The ROI score is only a heuristic used to select representative low-speed frames. The phrase boundary-sensitive velocity region refers to $\Omega_{\mathrm{ROI}}$. The phrase partially occluded or behind an active target refers to a candidate at similar bearing but larger range than an already active track, so the higher SNR threshold $\Gamma_{\mathrm{obs}}$ is applied during initiation.

The velocity axes in the RD figures use FFT-shifted radial-velocity bin centers converted with the chirp repetition interval and 24 GHz wavelength. They are physical Doppler-bin labels within the unambiguous radial-velocity interval, not vehicle ground-speed labels. Negative values follow the selected approaching-target sign convention used by the processing scripts. If a target exceeded the unambiguous radial-velocity range, the displayed bin would be aliased; this was not the intended operating condition of the low-speed bicycle and small-vehicle tests.

### 4.3. Labeled Synthetic Detection Evaluation

To address the absence of positive and negative labels in the natural measurement sequences, a labeled synthetic range–Doppler benchmark was added. Each trial generates a 64×32 RD power map with exponentially distributed clutter, a weak Doppler-boundary trend, and one compact moving-target response. The positive samples are the RD cells whose target component exceeds 22% of the injected target peak. All remaining RD cells are treated as negative samples. This definition gives complete cell-level labels, and therefore, supports standard detection metrics that cannot be obtained from the authors’ unlabeled natural scenes without additional annotation. The natural-data detection figures are, therefore, used only as representative boundary-behavior evidence, not as sequence-level precision, recall, F1, or ROC evidence.

**Figure 4:**
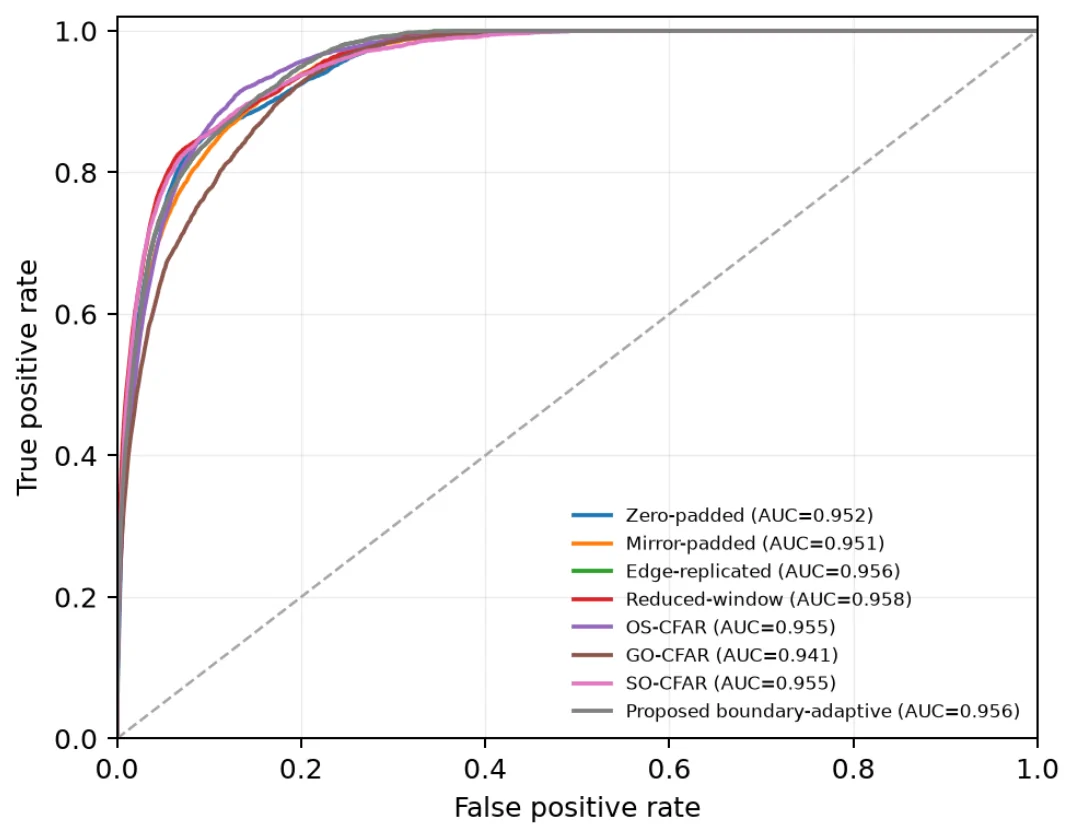
ROC curves for the labeled synthetic 64×32 RD benchmark. The curves are obtained by sweeping the continuous CUT-to-background score for each CFAR variant before applying the fixed binary threshold used in Table 2. The gray dotted diagonal denotes the random-classifier baseline.

All CFAR variants used the same 7×7 window, 5×5 guard region, 24 training cells, nominal $P_{\mathrm{fa}}=5\times 10^{-3}$, and 360 random trials generated with a fixed random seed. Here $P_{\mathrm{fa}}$ is a nominal threshold-control parameter because the frame-differenced magnitude statistic is not guaranteed to follow the exponential power distribution assumed by classical CA-CFAR. Precision, Recall, F1-score, and false alarms per frame were computed from cell-level true positives, false positives, false negatives, and true negatives. ROC curves, shown in Figure 4, were generated by sweeping the continuous CUT-to-background score before applying the binary CFAR threshold, and AUC was computed from the same labeled score distribution. The detection probability $P_{d}$ was counted as a target hit when at least one cell in a 3×3 neighborhood around the injected target center was detected. The complete script is eval_synthetic_detection_metrics.py.

The labeled benchmark changes the interpretation of the detection results. The proposed method is not presented as the highest-F1 detector. Zero padding and SO-CFAR give higher recall and $P_{d}$, but they also produce many more false alarms per frame. OS-CFAR and GO-CFAR reduce false alarms, but their recall and $P_{d}$ are substantially lower. The proposed boundary-adaptive CA-CFAR, therefore, occupies a middle operating point. Its main advantage is the boundary-sensitive $P_{d}$–false-alarm trade-off, not a universal maximum in F1-score. This supports the narrower claim that the proposed boundary treatment improves sensitivity without relying on the most permissive threshold relaxation.

**Table 2:** Standard labeled detection metrics on the synthetic 64×32 RD benchmark.

Method	Precision	Recall	F1	AUC	$P_{d}$	False Alarms per Frame
Zero-padded CA-CFAR	0.185	0.401	0.253	0.952	0.997	19.08
Mirror-padded CA-CFAR	0.339	0.199	0.251	0.951	0.778	4.19
Edge-replicated CA-CFAR	0.353	0.213	0.266	0.956	0.867	4.22
Reduced-window CA-CFAR	0.372	0.268	0.312	0.958	0.994	4.88
OS-CFAR	0.394	0.094	0.151	0.955	0.703	1.55
GO-CFAR	0.389	0.131	0.196	0.941	0.717	2.21
SO-CFAR	0.247	0.373	0.297	0.955	0.967	12.25
Proposed boundary-adaptive CA-CFAR	0.352	0.213	0.266	0.956	0.867	4.22

### 4.4. Detection Performance Experiments

The natural-data detection experiment is retained as a boundary-behavior illustration rather than as the primary detection metric. It was evaluated on the low-speed target sequence 0604_6.txt. Two representative frames were selected by a heuristic ROI response score computed from $N_{\mathrm{ROI}}$, the stage-to-stage ROI-count gains, and $C_{\mathrm{ROI}}$. The score is used only to choose visually representative frames and is not used as a standard detection metric. The authors’ natural bicycle and vehicle sequences do not have manually annotated positive and negative RD cells over all frames. Consequently, the manuscript does not claim sequence-level real-data Precision, Recall, F1-score, or ROC performance for the natural scenes. The boundary-sensitive velocity region denotes $\Omega_{\mathrm{ROI}}$, where the CFAR training window is most affected by invalid Doppler-side samples. The selected raw frames are frame 378 at 18.900 s and frame 866 at 43.300 s. The experiment compares two processing factors: intra-frame differencing versus frame-differential RD enhancement, and mirror padding versus the proposed boundary-adaptive spectral extension for CA-CFAR threshold estimation.

To separate the effect of each detection-stage change, the same two raw frames were also processed with a module-level ablation. The ablation keeps the CFAR mask, guard region, and $P_{\mathrm{fa}}$ fixed, and changes only the RD preprocessing and boundary treatment. Frame-differential RD enhancement with mirror padding increases the ROI detections from 5 to 14 in frame 378 and from 12 to 16 in frame 866, while boundary-adaptive CA-CFAR on the raw RD map increases them to 14 and 19. The full detection chain reaches 23 ROI detections in both frames, indicating that the useful gain comes from coupling motion-sensitive RD enhancement with boundary-stable CFAR rather than from a single looser threshold.

The same two frame-differential RD maps were also processed with several CFAR boundary and statistic variants. All variants used the same 7×7 mask, guard region, and nominal $P_{\mathrm{fa}}$. The baselines were zero padding, mirror padding, edge replication, reduced-window CA-CFAR, OS-CFAR, GO-CFAR, and SO-CFAR. The reduced-window rule drops invalid training cells near the Doppler edge and computes the CA estimate from the remaining valid cells. The comparison used ROI detections, top-ROI hits, detected-ROI margin, and boundary-threshold CV. The proposed padding rule is interpreted as a scenario-conditioned boundary treatment for this retained quadrant, not as a generally CFAR-optimal padding rule.

**Figure 5:**
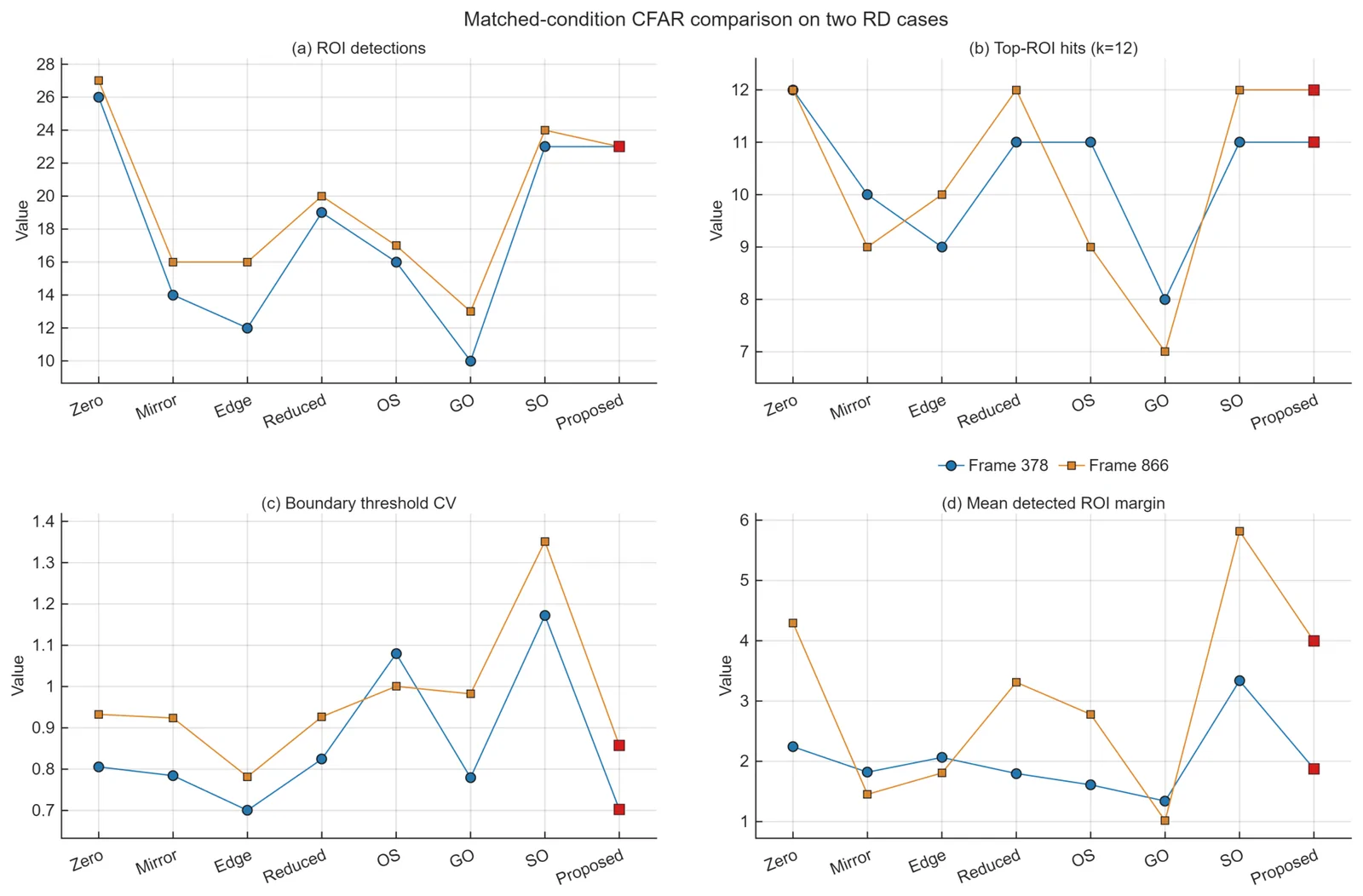
Matched-condition CFAR comparison on two representative low-speed RD cases. The same CFAR mask, guard region, and nominal false-alarm probability were used for all boundary and statistic variants. Red squares mark the proposed boundary-adaptive CA-CFAR values.

The expanded CFAR-variant comparison is summarized in Figure 5. Zero padding gives the largest ROI counts and reaches all 12 manually selected top-ROI cells in both frames, but it follows a permissive boundary pattern. SO-CFAR behaves similarly: it retains 23 and 24 ROI detections and reaches 11 and 12 top-ROI hits, but it also gives the largest detected-ROI margin and the highest boundary-threshold CV. GO-CFAR is the most conservative baseline, with 10 and 13 ROI detections and 8 and 7 top-ROI hits in the two frames. Mirror padding, edge replication, and OS-CFAR occupy the middle range but miss part of the strongest boundary-adjacent response. The reduced-window baseline improves sensitivity relative to mirror padding and reaches all 12 top-ROI cells in frame 866. Its boundary-threshold CV remains above the proposed method in both frames. The proposed boundary-adaptive CA-CFAR is not the maximum-ROI method, but it preserves 23 ROI detections in both frames. It also reaches 11 and 12 top-ROI hits while keeping the boundary-threshold CV below mirror padding, reduced-window CA-CFAR, OS-CFAR, and SO-CFAR.

As a numerical robustness check, a synthetic quarter-RD Monte-Carlo boundary test was also constructed. Each trial contains exponentially distributed clutter with a weak boundary trend and one low-speed target. The target was modeled as a compact Gaussian response placed 0–3 Doppler cells away from the processed boundary. The sweep uses 240 random trials per distance and reports detection probability, false alarms per frame, and boundary-threshold CV under the same CFAR mask and nominal $P_{\mathrm{fa}}$.

**Figure 6:**
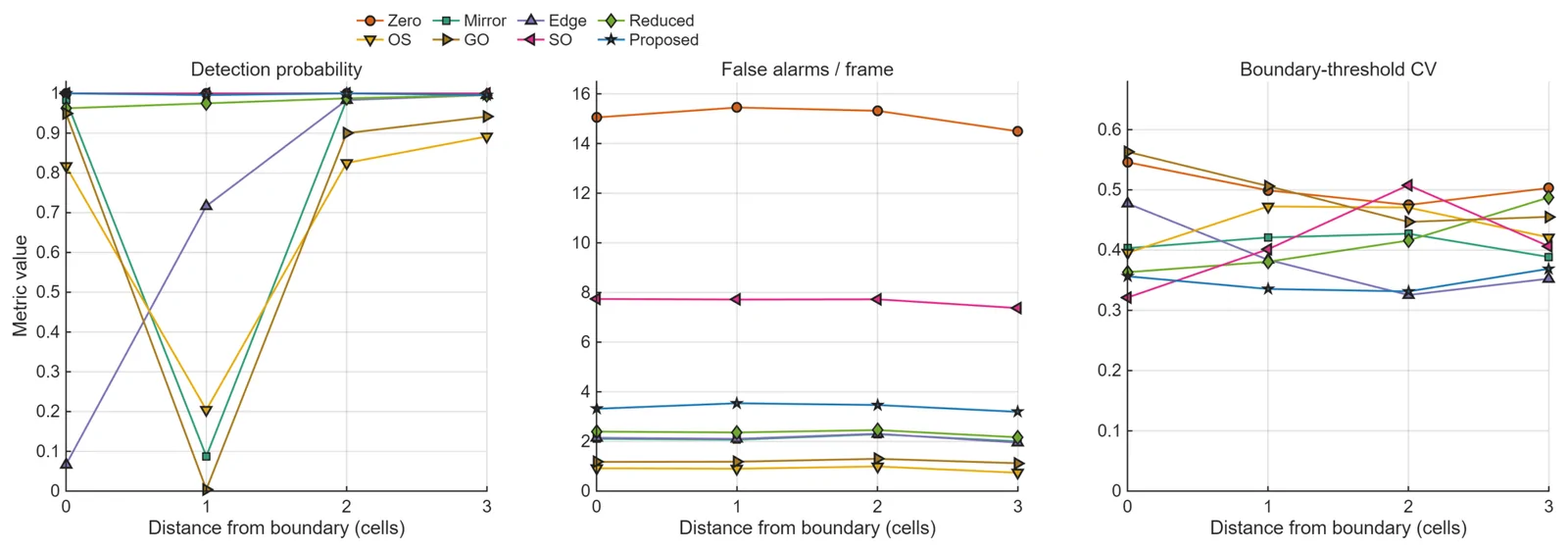
Synthetic quarter-RD boundary sweep for CFAR variants. Each point summarizes 240 Monte-Carlo trials at a specified target distance from the processed Doppler boundary.

The sweep result is shown in Figure 6. At one cell from the boundary, the proposed method keeps $P_{d}=0.996$. Mirror padding drops to 0.088, OS-CFAR to 0.204, and GO-CFAR to 0.004. Zero padding and SO-CFAR also keep $P_{d}=1$, but their mean false-alarm counts increase to 15.45 and 7.72 per frame. The corresponding value for the proposed method is 3.54 per frame. Across distances 0–3, the proposed boundary-threshold CV remains between 0.331 and 0.369. OS-CFAR yields fewer false alarms than the proposed rule, but with lower boundary-adjacent detection probability.

**Figure 7:**
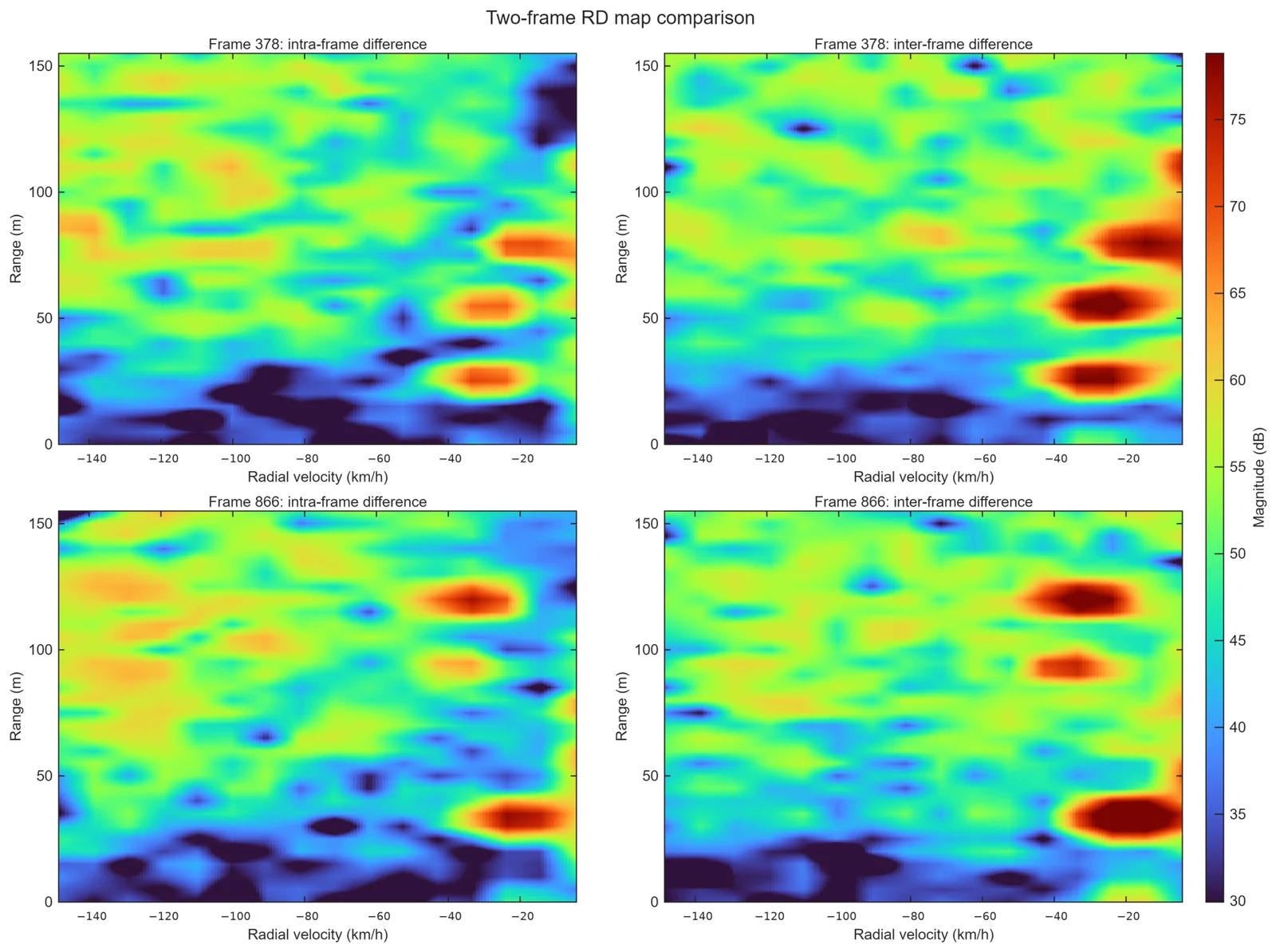
RD-map comparison between intra-frame differencing and frame-differential RD enhancement for two representative low-speed frames. All panels use the same range–Doppler bin layout exported by the processing script. The frame-differential maps preserve the moving low-speed response while attenuating slowly varying background components.

Figure 7 shows that frame-differential RD enhancement suppresses the static and slowly varying background while keeping the low-speed moving response concentrated. This cleaner RD map reduces the bias of the CA-CFAR training cells before thresholding.

**Figure 8:**
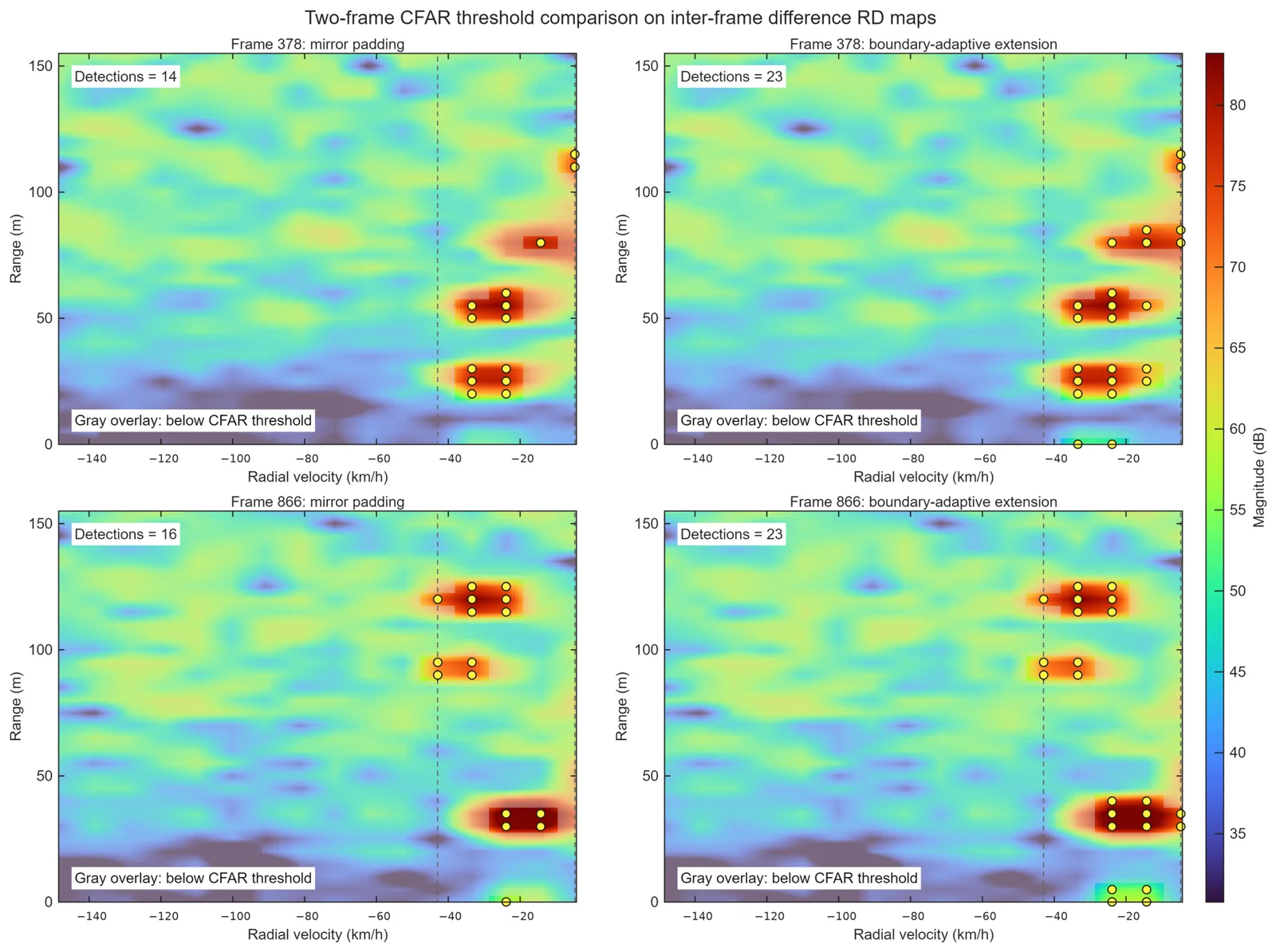
CFAR detection comparison on frame-differential RD maps. Yellow markers denote cells above the CA-CFAR threshold, and the gray overlay denotes cells below the threshold. The dashed vertical lines mark the last five Doppler columns defining the boundary-sensitive ROI $\Omega_{\mathrm{ROI}}$.

Figure 8 gives the representative baseline result. In frame 378, the full chain produces 23 ROI detections, compared with 8 for the intra-frame-differencing and mirror-padding baseline. In frame 866, the count is again 23, compared with 10 for the same baseline. The improvement comes from two linked factors: frame-differential RD enhancement increases target-to-background contrast, and boundary-adaptive extension keeps the CFAR reference window complete near the useful velocity boundary.

### 4.5. Seed-Growing Aggregation Versus DBSCAN

**Figure 9:**
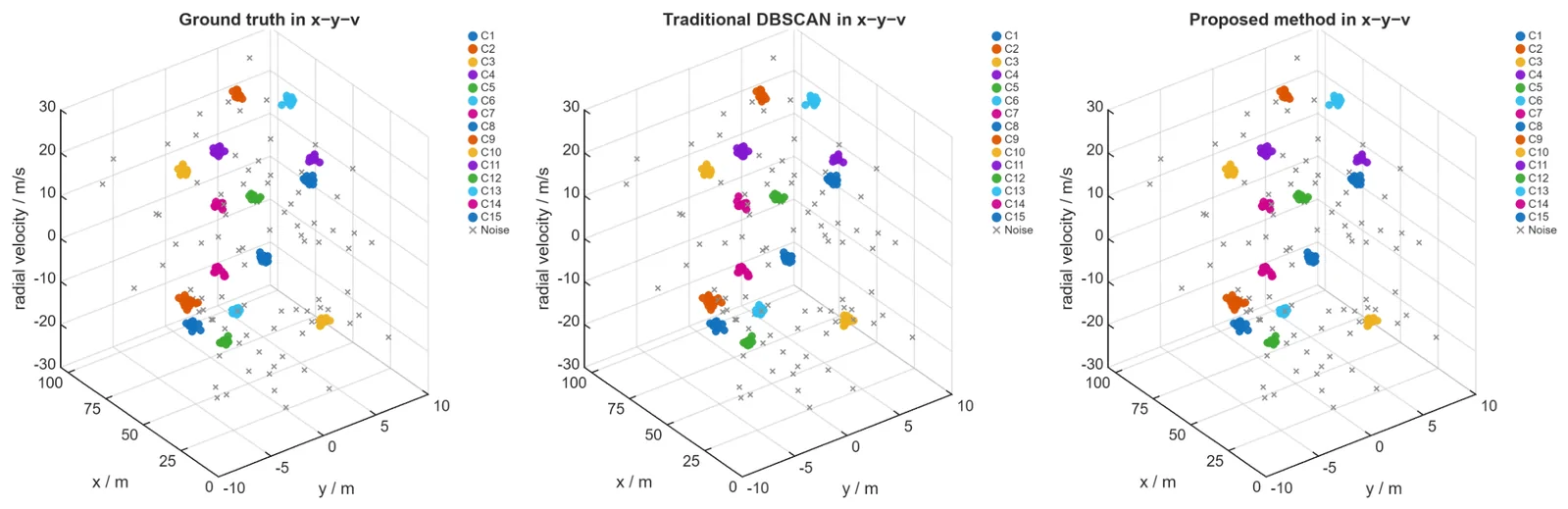
Comparison of target point aggregation in the simulated *x*-*y*-$v_{r}$ space. The axes are the simulated Cartesian position and radial-velocity coordinates generated by the parameter-sweep script. The left panel shows the ground truth, the middle panel shows the conventional DBSCAN result in normalized position–velocity space, and the right panel shows the proposed seed-growing result. Gray markers denote clutter or rejected points.

The proposed target-initiation module was further compared with a conventional DBSCAN clustering baseline using the simulation data in Figure 9. The simulated point clouds contain 15 traffic-like targets, 10–28 radar plots per target, range-dependent SNR attenuation, radial-velocity dispersion, and 120 clutter points with static, medium-speed, and high-speed components. This setting stresses a common radar-tracking difficulty: the target point density, SNR, and radial velocity are not uniform across range and lane position, whereas a standard DBSCAN implementation must still rely on a single normalized distance scale and a single core-point density threshold.

For the DBSCAN baseline, clustering was performed in the normalized *x*-*y*-$v_{r}$ feature space,

(48) $$z_{i}^{\mathrm{DB}}={\left[\frac{x_{i}}{\tau_{d}^{\mathrm{DB}}},\frac{y_{i}}{\tau_{d}^{\mathrm{DB}}},\frac{v_{r,i}}{\tau_{v}^{\mathrm{DB}}}\right]}^{T},\;{∥z_{i}^{\mathrm{DB}}-z_{j}^{\mathrm{DB}}∥}_{2}\leq \epsilon ,$$

where ϵ and $N_{\min}$ define the density neighborhood. In contrast, the proposed method follows the seed-growing validity tests introduced in Section 3.3. Spatial compactness, radial-velocity consistency, effective motion, and SNR reliability are kept as separate physical decisions rather than being compressed into one Euclidean density parameter. This distinction is important for embedded radar processing because each threshold corresponds to a measurable radar quantity and can be bounded without a global neighborhood search.

The Monte-Carlo comparison used 100 random runs. Both methods associated all 15 targets on average and produced almost identical target purity. The main difference was computational: seed-growing reduced the MATLAB R2025b runtime from 3.248 ms to 2.334 ms, a 28.1% reduction, because it avoids the explicit pairwise distance matrix used by DBSCAN.

Parameter sensitivity was further evaluated with 320 DBSCAN configurations and 144 seed-growing configurations. The sweep used an empirical ranking score defined in this work as $J=N_{\mathrm{pure}}-1.3N_{\mathrm{merge}}-N_{\mathrm{false}}-1.1N_{\mathrm{miss}}+0.4R_{\mathrm{rej}}$, where $N_{\mathrm{pure}}$, $N_{\mathrm{merge}}$, $N_{\mathrm{false}}$, $N_{\mathrm{miss}}$, and $R_{\mathrm{rej}}$ denote correctly separated targets, merged clusters, false clusters, missed targets, and clutter rejection ratio. The weights are not taken from a standard clustering metric; they encode the experiment priority used here: merged targets and missed targets are penalized more strongly than isolated clutter-dominated clusters, while clutter rejection is used as a secondary tie-breaker.

The best-parameter sweep gave very similar top-end results for the two clustering approaches: DBSCAN reached its best score at ϵ=0.95, $N_{\min}=4$, $\tau_{d}^{\mathrm{DB}}=1.4$, and $\tau_{v}^{\mathrm{DB}}=1.0$, while seed-growing reached its best score at $\tau_{d}=1.4$, $\tau_{v}=1.5$, $N_{\min}=4$, and $\Gamma_{\min}=7.5$. Both best configurations achieved complete target association with no false or merged clusters. DBSCAN remains more general geometrically, but its density parameters are coupled. Seed-growing is narrower, yet better matched to compact radar plots with coherent radial velocity. In the sweep, increasing $N_{\min}$ from 2 to 4 reduced the average false-cluster count from 1.508 to 0.016, and increasing $\Gamma_{\min}$ from 5.5 to 7.5 reduced it from 0.986 to 0.171. This supports using physically separated motion and SNR gates for embedded target initiation.

### 4.6. Tracking Performance Experiments

Tracking performance was evaluated on natural measurement data from two scenes: a bicycle single-target scene and a small-vehicle multi-target scene. The proposed finite-frame retained Kalman strategy was compared with an immediate-deletion baseline. The proposed strategy retains the predicted state for a limited number of missed frames. A track can, therefore, survive short measurement gaps and continue with the same identity when a valid measurement returns.

The tracking comparison uses radar-derived same-sequence continuity metrics. The reported position error is the average Cartesian residual computed from the radar-derived reference trajectory and the associated track state in the evaluation script. ID-switch counts are computed by nearest-track identity changes in the available radar trajectories. These metrics compare lifecycle strategies on the same data stream.

For reproducibility, the natural-scene continuity metrics are defined on the radar-derived trajectory sequence. Let $r_{k}={\left[x_{k}^{\mathrm{ref}},y_{k}^{\mathrm{ref}}\right]}^{⊤}$ be the selected radar-derived reference position at frame *k*, and let $s_{j,k}={\left[x_{j,k},y_{j,k}\right]}^{⊤}$ be the Cartesian position of output track *j*. The associated track index and Cartesian residual are

(49) $$j_{k}^{★}=\arg\min_{j}∥s_{j,k}-r_{k}{∥}_{2},\;e_{k}={∥s_{j_{k}^{★},k}-r_{k}∥}_{2}.$$

The reported position error is $\bar{e}=K^{-1}\sum_{k=1}^{K}e_{k}$. The velocity standard deviation is computed from the associated track radial-velocity samples over the same valid frames. A track break is counted when no associated track is present for an otherwise valid reference interval, and an ID switch is counted when $j_{k}^{★}\neq j_{k-1}^{★}$ across consecutive valid frames.

The MATLAB real-data tracking evaluation uses the same fixed-capacity structure as the project tracker: at most 20 tracks, at most 100 input points, and a 2D constant-acceleration state with Δt=0.05s. The numerical thresholds differ from the representative settings in Table 1 because this evaluation uses the exported MATLAB plot-amplitude scale rather than the compact firmware demonstration scale. The values used for the natural-scene tracking metrics are as follows: maximum radial velocity 41.1m/s, radial-velocity resolution 2.5699m/s, stored acceleration-limit vector $\left[-0.1,-0.1,-0.1\right]\;m/s^{2}$ with covariance based on $|a_{\max}|=0.1\;m/s^{2}$, allocation thresholds $\left[\Gamma_{\mathrm{normal}},\Gamma_{\mathrm{obs}},v_{\min},N_{\min},\tau_{d},\tau_{v},v_{\mathrm{static}}\right]=\left[4000,4000,3,2,10,6,0\right]$, scene limits [1,−3,3,0,160,−2.5,2.5,0], and lifecycle thresholds $\left[T_{\mathrm{act}},T_{\det},T_{\mathrm{free}},T_{\mathrm{static}},T_{\mathrm{exit}},T_{\mathrm{sleep}}\right]=\left[6,6,10,30,20,10\right]$ frames. The gating vector is [9,15,0,0,8], the variation vector is [0.5,0.5,0.3,0.3], the velocity unrolling uses smoothing factor 0.3 and confidence threshold 0.15, and the 2D tracker starts from minimum spread $\left[0.25,1^{\circ },0.25\right]$, initial spread $\left[0.5,2^{\circ },0.5\right]$, and $P_{0|0}=\mathrm{diag}\left(0,0,0.5,0.5,1,1\right)$. These values, rather than the representative values in Table 1, define the natural-data tracking evaluation setting.

**Figure 10:**
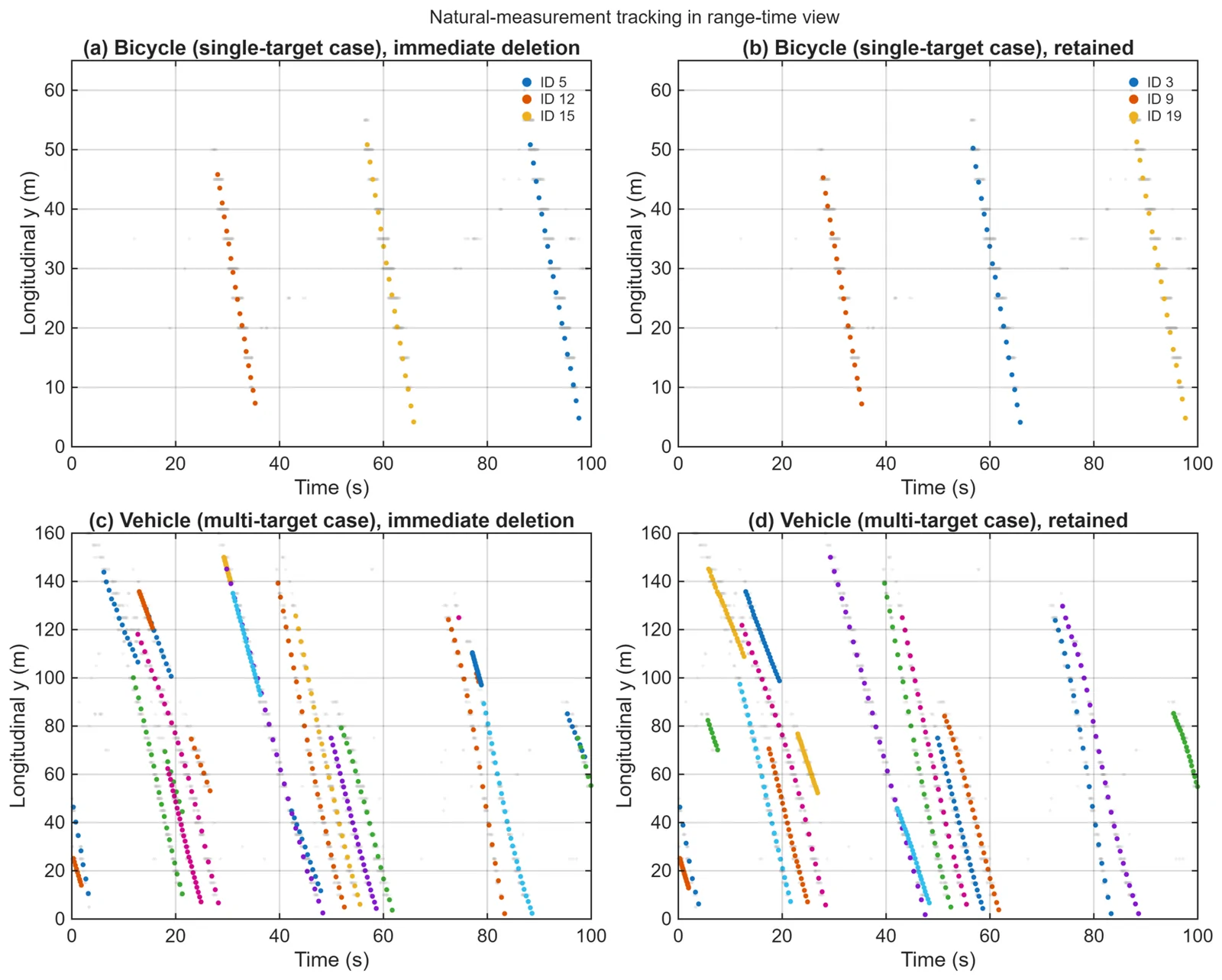
Tracking results on natural measurement data shown in range–time view. Panels (**a**,**b**) compare the immediate-deletion baseline and finite-frame retained Kalman tracking in the bicycle single-target scene. Panels (**c**,**d**) compare the same two lifecycle strategies in the small-vehicle multi-target scene. Gray markers show masked radar measurements, and colored markers show tracker-output states grouped by tracker ID.

Figure 10 summarizes the natural-data tracking comparison as a range–time measurement and track overlay. In the bicycle scene, finite-frame retained Kalman tracking extends the continuous tracking length from 188 to 201 frames while slightly reducing the velocity standard deviation from 0.144 m/s to 0.134 m/s, with little change in the radar-derived position residual (1.707 m versus 1.718 m). In the vehicle scene, the benefit is much clearer: ID switches are reduced from 74 to 5, the average position residual decreases from 1.431 m to 1.331 m, the velocity standard deviation decreases from 0.985 m/s to 0.728 m/s, and the associated points per frame remain similar (2.995 versus 3.066).

An additional controlled short-miss experiment was generated from the same real radar measurements by masking points around a radar-derived reference track for a short interval and then comparing finite-frame retention with immediate deletion. This controlled gap directly tests the retained Kalman state machine under a known measurement interruption. Table 3 shows that finite-frame retention preserves the interrupted track identity in both scenes, whereas immediate deletion fails the reassociation test and produces many more track interruptions.

### 4.7. Public Radar Benchmark Evaluation

To address the reviewers’ request for radar-specific tracking metrics and a classical multi-target tracking baseline, the public-radar evaluation was rerun on three public resources: RadarScenes, the public RADIATE foggy sample, and nuScenes mini radar-only sequences [16,17,18]. The purpose of this benchmark is deliberately narrower than the board-level experiment. These public data provide labeled radar point centers, annotated radar-image centers, or radar detections with object annotations, but they do not provide the present board’s 64×32 RD frames, boundary-affected CA-CFAR maps, or board-specific SNR fields. The public benchmark, therefore, evaluates the finite-frame retained lifecycle and data-association behavior under common public observations. It is a tracker-lifecycle evaluation, not a full-pipeline validation of the authors’ board-specific processing chain. It does not claim to reproduce the full frame-differential RD enhancement, boundary-adaptive CFAR, seed-growing, and SNR-weighted centroid pipeline used on the authors’ 24 GHz board.

Within each dataset, all trackers used the same observations, temporal order, match threshold, and metric implementation. The compared methods were the proposed finite-frame retained lifecycle adapter and a bounded-approximation JPDA baseline. The finite-hold adapter used a constant-velocity nearest-neighbor update and retained unmatched tracks for a tuned number of frames. The JPDA baseline used the same motion model and gate family, enumerated joint association events for small gated components, and used a bounded mutually exclusive approximation when the gated component became too large. RadarScenes was evaluated from public track-labeled radar points after grouping non-static points by public track ID at each timestamp, giving one pre-clustered target-center observation per labeled radar target. These target centers were derived from public labels only to isolate the tracker lifecycle. No claim is made for raw radar point-cloud clustering performance in this public-data experiment. MOTA, MOTP, IDF1, ID switches, fragmentation, Precision, and Recall were computed with a center-distance greedy MOT-style protocol; the RadarScenes match threshold was 2.5 m. Parameters were selected by a dataset-specific grid search on the evaluated subset; the final values are reported in Table 4. Runtime reports the tracker update only and excludes data loading, file output, plotting, and parameter sweeping.

The filtered multi-subset results are retained under radarscenes_track_center_input_metrics and public_radar_finite_hold_multi_dataset_no_immediate. The sweep includes four independent RadarScenes sequences, four RADIATE foggy prefixes (200, 400, 600, and all available frames), and three nuScenes mini settings (2 scenes, 5 scenes, and all 10 mini scenes). Table 5 reports the four RadarScenes sequences together with one representative RADIATE adverse-weather subset and one sparse nuScenes mini radar-only subset.

**Figure 11:**
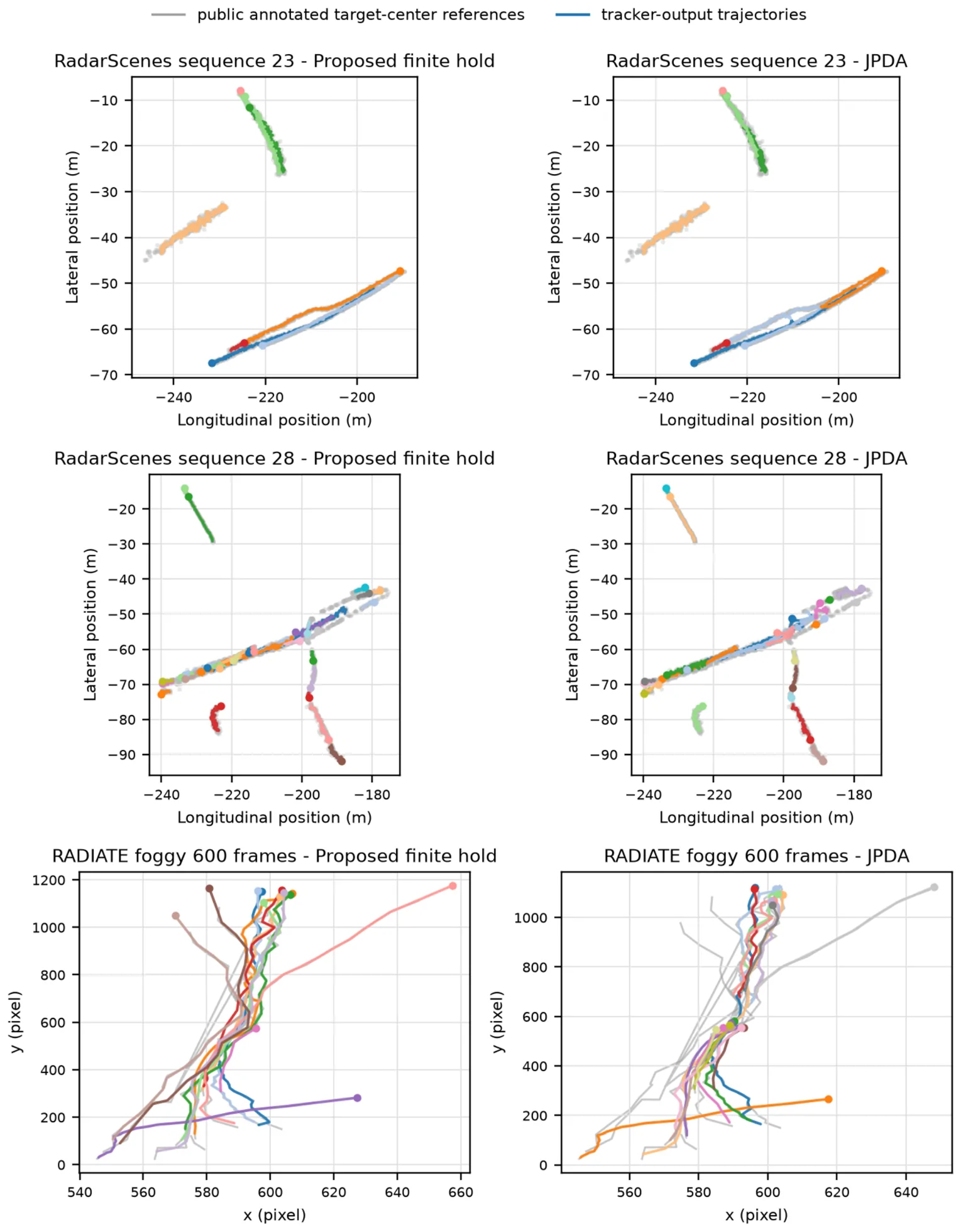
Selected public-radar trajectory examples for the proposed finite-hold tracker and the bounded-approximation JPDA baseline. The first two rows use RadarScenes target-center observations derived from non-static radar points grouped by public track ID; gray markers denote the public target-center references and colored curves denote tracker-output trajectories. The third row overlays RADIATE foggy tracker outputs with public radar-image bounding-box centers used as annotated target-center references. These visual examples are paired with the standard MOTA, MOTP, IDF1, identity-switch, fragmentation, Precision, Recall, and runtime values in Table 5.

Figure 11 provides the public-data trajectory visualization retained in the main text. It includes RadarScenes sequence 23 as a cleaner target-center case, RadarScenes sequence 28 as a more fragmented case, and the RADIATE foggy sample as an adverse-weather radar-image case. The RadarScenes panels show target-center references derived from public track annotations in gray and tracker-output trajectories in color. The RADIATE panels similarly overlay the public radar-image bounding-box centers as annotated target-center references, so the adverse-weather example is not shown as tracker output alone. The sparse nuScenes plots are omitted from the visual evidence and retained only in the metric table as a boundary case.

On RadarScenes, the input observations were the mean sequence-coordinate positions of non-static radar points grouped by the public track ID at each timestamp. Across the four evaluated sequences, the proposed finite-hold tracker obtained MOTA values between 0.878 and 0.968 and IDF1 values between 0.670 and 0.959. The bounded-approximation JPDA baseline was comparable on sequence 25 and slightly higher in IDF1 on the more fragmented sequence 28, but it produced more identity switches on sequences 10, 23, and 28 and required longer tracker-update time. The average tracker-update time was 5.87 ms for the finite-hold adapter and 83.52 ms for the JPDA baseline over the four RadarScenes sequences. These results support the intended embedded claim: the proposed lifecycle rule provides competitive target-center tracking with lower computational cost, rather than replacing probabilistic association in all scenes.

The RADIATE foggy sample was used as an adverse-weather radar-image case. The tracker input was the center of each annotated radar-image bounding box. Under the tuned setting, the proposed tracker reached higher IDF1 than the bounded-approximation JPDA baseline (0.907 versus 0.688), produced fewer ID switches (1 versus 27), and slightly higher recall (0.963 versus 0.932). This result should not be read as universal superiority over probabilistic association. It indicates that finite retention is useful when the dominant failure mode is short target disappearance in compact target-center sequences.

The nuScenes mini experiment was retained as a radar-only stress test rather than as a full nuScenes tracking benchmark submission. Radar points from the mini split were associated with object annotations in the radar frame, without camera, lidar, map, or learned detector inputs. Under this deliberately sparse input, both methods produced negative MOTA because false tracks and missed objects were frequent. The proposed finite-hold adapter slightly exceeded the bounded-approximation JPDA baseline in IDF1 (0.095 versus 0.092) and MOTA (−1.026 versus −1.147), whereas the JPDA baseline gave marginally higher recall (0.261 versus 0.260). This result defines the boundary of the lifecycle rule: finite retention helps short-gap continuity, but it cannot compensate for weak radar-only object evidence in a full urban-driving benchmark.

Taken together, the public radar results replace the earlier ROI-count-only tracking evidence with standard metrics and a classical multi-object tracking comparator. They also bound the claim. After dataset-specific tuning, the finite-frame retained lifecycle rule is much faster than the bounded-approximation JPDA baseline on all four RadarScenes sequences and gives stronger identity continuity on the RADIATE foggy sample. On nuScenes mini, however, radar-only input remains too sparse for either compared tracker to produce strong MOTA. The public-data result should, therefore, be read as tracker-lifecycle evidence for short-gap retention, not as a claim of universal dominance over JPDA or as a full validation of the board-specific RD-to-track pipeline.

### 4.8. Real-Time Analysis

Because this study is oriented toward embedded platforms, runtime was analyzed in the same order as the firmware data path. The stages were frame acquisition and unpacking, radar signal processing, plot compaction, seed-growing target initiation, Kalman tracking, and UART packet output. The timing analysis was performed with the project C implementation in a host-side firmware-path model, not from direct cycle logs recorded on the STM32F405 board. The timing script uses a fixed 64×32 frame format, the STM32F405RGT6-oriented processing path, and the same fixed-capacity design used by the detection and tracking modules. The range–Doppler grid, intermediate buffers, target arrays, and UART packets are dimensioned statically rather than by dynamic allocation.

In the current timing model, frame acquisition requires 2.668 ms, data unpacking 0.245 ms, the RD/CFAR processing block 5.702 ms, and UART output 0.472 ms. The remaining target-dependent stage includes plot compaction, seed-growing initiation, tracking update, and packet packing. Its runtime ranges from 0.102 to 5.809 ms as the scene load increases. Table 6 reports the resulting full-frame runtime under different target loads.

The no-plot full-frame runtime is 9.189 ms, or 18.4% of the 50 ms frame period at 20 Hz. Runtime then increases approximately with the plot count because seed-growing, target-array construction, association, and packet preparation operate on compacted plots rather than on the full range–Doppler grid. At the largest tested load, 12 tracks and 48 plots, the target-dependent stage reaches 5.809 ms, the full-frame mean reaches 14.896 ms, and the worst observed full-frame value is 15.837 ms, or 31.7% of the frame period. These modeled values support the plausibility of the fixed-size embedded design under the tested frame format, but they are not presented as direct on-board timing validation. A stronger hardware-validation claim would still require board timing logs, compiled memory reports, and the exact build configuration.

## 5. Discussion

The experiments support a bounded implementation claim rather than a general radar-perception benchmark claim. The strongest evidence is the matched-condition improvement of the specific weak points observed on the compact board: static clutter leakage, incomplete CFAR reference windows near the processed Doppler boundary, unstable target initiation from sparse plots, and short echo gaps that would otherwise break track continuity. The natural-data detection figures illustrate these boundary effects in representative frames; they are not used as sequence-level labeled detection metrics.

The main interpretive risk is that some gains could be attributed to a looser decision threshold rather than to better signal conditioning. The added module ablation, multi-baseline CFAR comparison, and synthetic boundary sweep reduce that concern by separating RD enhancement from boundary-threshold construction and by reporting both sensitivity-oriented and stability-oriented indicators. The contribution should, therefore, be read as a deterministic, parameter-explicit processing chain for a low-cost FMCW board, not as a claim of broad superiority across radar modalities, datasets, or tracking paradigms.

The added labeled synthetic benchmark reduces the detection-metric gap by supplying complete positive and negative RD-cell labels for Precision, Recall, F1-score, ROC/AUC, $P_{d}$, and false-alarm reporting. The public-radar runs further address the tracking-metric gap by reporting MOTA, MOTP, IDF1, ID switches, fragmentation, Precision, Recall, and runtime on RadarScenes, the public RADIATE foggy sample, and nuScenes mini radar-only sequences. These public radar results evaluate tracker-level behavior under common target-center observations. They do not validate the full board-specific RD-to-track chain, which is evaluated only on the authors’ compact-platform cases.

These results also expose an important boundary. The proposed finite-frame rule is comparable to the bounded-approximation JPDA baseline across multiple RadarScenes sequences at lower computational cost. It gives stronger identity continuity on the selected RADIATE foggy subset when the dominant failure mode is short target disappearance. On nuScenes mini, however, sparse radar-only detections lead to negative MOTA for both compared trackers. Lifecycle retention alone cannot replace stronger detection evidence or multi-modal tracking in that setting. The method should, therefore, be interpreted as an embedded lifecycle strategy whose advantage depends on correct measurement modeling and short-gap-dominated failure modes.

## 6. Conclusions

This work examined a complete FMCW radar detection and tracking chain for low-speed bicycle and small-vehicle sensing on a compact STM32F405-based 24 GHz board. The implemented chain keeps the classical range–Doppler, CA-CFAR, and Kalman-filter backbone, but strengthens the stages that were weakest in the measured data: spectral-domain background suppression, CFAR behavior at the processed Doppler boundary, target initiation from sparse plots, and track survival after short echo gaps.

Under matched low-speed conditions, the added evidence shows that the gain is not explained by a single aggressive boundary rule. The full chain improves boundary-sensitive detections in representative RD frames, while sequence-level labeled detection metrics are reported only on the synthetic RD benchmark. The expanded MATLAB CFAR comparison clarifies the sensitivity–stability trade-off among zero, mirror, edge, reduced-window, OS, GO, SO, and the proposed rule. The labeled synthetic RD benchmark reports standard Precision, Recall, F1-score, ROC/AUC, $P_{d}$, and false-alarm statistics.

On the tracking side, seed-growing remains competitive with tuned DBSCAN under the simulated workload. Finite-frame retained Kalman tracking improves same-sequence continuity relative to immediate deletion in both natural and controlled short-gap tests. Public-radar evaluation on RadarScenes, the RADIATE foggy sample, and nuScenes mini radar-only sequences reports MOTA, MOTP, IDF1, ID switches, fragmentation, Precision, Recall, runtime, and comparisons with a bounded-approximation JPDA baseline. These public results evaluate tracker-lifecycle behavior, whereas the complete board-specific RD-to-track chain is evaluated only on the authors’ compact-platform cases. The proposed tracker is comparable to the JPDA baseline across multiple RadarScenes sequences at much lower runtime and gives higher IDF1 with fewer ID switches on the selected RADIATE foggy subset. The nuScenes mini result is retained as a boundary case for sparse radar-only urban tracking, not as evidence of general benchmark dominance. In the host-side firmware-path timing model, even the largest tested load stays below 15.837 ms, leaving margin within the 50 ms frame period at 20 Hz on an STM32F405RGT6-class controller.

The claim remains deliberately narrow. The tested platform uses a 24 GHz carrier and 30 MHz modulation bandwidth, so its nominal range resolution is only about 5.0 m and its Doppler-bin spacing is about 2.57 m/s. Its angular performance is also limited by the compact board antenna and the absence of a complete angle-calibration record in the current project materials. Therefore, the method should be interpreted as a practical deterministic processing chain for this low-resolution embedded board and the tested low-speed scenes. Its applicability does not extend to high-resolution imaging radar or high-angular-resolution MIMO radar systems without separate validation, retuning, and benchmark evaluation. Future work should expand the public-dataset evaluation to full benchmark splits and additional adverse scenarios, improve calibration, and report direct MCU memory and timing measurements.

## Figures and Tables

**Table 1:** Experimental platform and representative board-level parameter settings. Tracking parameters used for the natural-scene continuity test and public radar benchmark are reported separately in the corresponding evaluation subsections.

Category	Parameter	Value or Description
Hardware platform	Radar board	Compact 24 GHz FMCW PCB with STM32F405RGT6 MCU, USB Type-C interface, UART header, and planar antenna side
Hardware platform	MCU resources	ARM Cortex-M4F at up to 168 MHz, 1 MB Flash, 192 KB SRAM, single-precision FPU
Hardware platform	Approximate PCB scale	About 5 cm class, estimated from the ruler shown in Figure 3
Radar parameter	Carrier frequency	24 GHz
Radar parameter	Modulation bandwidth	30 MHz
Radar parameter	Sampling rate	1 MHz
Radar parameter	Nominal resolution	ΔR=5.0m, Doppler-bin spacing ≈2.57m/s
Frame structure	Chirps per frame	32
Frame structure	Samples per chirp	64 valid I/Q samples
Frame structure	Frame rate	20 Hz
Coordinate convention	Doppler axis	FFT-shifted radial-velocity bin centers; negative velocity denotes the selected approaching-target sign convention in the retained quadrant
Signal processing	Range-FFT length	64
Signal processing	Doppler-FFT length	32
Detection algorithm	RD enhancement	Frame-differential RD enhancement in the target-relevant quadrant
Detection algorithm	CFAR type	Boundary-adaptive CA-CFAR with 7×7 window, 5×5 guard region, 24 training cells, and nominal $P_{\mathrm{fa}}=5\times 10^{-3}$
Track initiation	Plot aggregation	Representative seed-growing setting with $\Gamma_{\mathrm{normal}}=80$, $\Gamma_{\mathrm{obs}}=80$, $v_{\min}=3\;m/s$, $N_{\min}=3$, $\tau_{d}=10\;m$, $\tau_{v}=6\;m/s$, and $v_{\mathrm{static}}=0.2\;m/s$
Tracking algorithm	Filtering model	Constant-acceleration Kalman prediction with SNR-weighted measurement update
Tracking algorithm	Gate and retention	Gate gain 9; measurement limits 20m, 10m, 0m, 15m/s; lifecycle thresholds $T_{\mathrm{act}}=5$, $T_{\det}=5$, $T_{\mathrm{free}}=20$ frames

**Table 3:** Controlled short-miss tracking comparison from real radar measurements.

Scene	Strategy	Continuous Frames	Reassoc. Success	Track Breaks	Velocity Std. (m/s)	Occlusion Residual (m)
Bicycle	Finite-frame retained Kalman	213	1/1	3	0.133	0.021
	Immediate deletion	66	0/1	18	0.114	0.941
Vehicle	Finite-frame retained Kalman	375	1/1	4	0.729	0.009
	Immediate deletion	134	0/1	37	1.353	0.313

**Table 4:** Selected tracking parameters after dataset-specific grid search. Gate and match units follow the corresponding dataset coordinate system. A dash indicates that the parameter is not used by that tracker.

Dataset Subset	Tracker	Gate	Max Missed	JPDA σ/Gate	$P_{D}$	Assignment Prob.
RadarScenes sequences	Proposed finite hold	5.5 m	5	–	–	–
RadarScenes sequences	JPDA	5.5 m	5	0.35	0.90	0.05
RADIATE foggy 600 frames	Proposed finite hold	80 px	3	–	–	–
RADIATE foggy 600 frames	JPDA	60 px	1	0.35	0.98	0.35
nuScenes mini all scenes	Proposed finite hold	6.5 m	1	–	–	–
nuScenes mini all scenes	JPDA	5.0 m	1	–	–	–

**Table 5:** Public radar tracker-lifecycle metrics on representative public subsets. RadarScenes and nuScenes values are reported in meters; RADIATE values are reported in radar-image pixels. Runtime reports tracker-update time only, excluding dataset loading, plotting, and parameter search.

Dataset Subset	Tracker	MOTA	MOTP (m/px)	IDF1	IDS	Frag.	Prec.	Recall	Runtime (ms)
RadarScenes seq. 10, 1000 frames	Proposed finite hold	0.911	0.346 m	0.882	15	39	1.000	0.916	7.90
RadarScenes seq. 10, 1000 frames	JPDA	0.837	0.380 m	0.826	89	49	0.950	0.913	104.93
RadarScenes seq. 23, 1000 frames	Proposed finite hold	0.968	0.156 m	0.959	4	10	1.000	0.970	6.08
RadarScenes seq. 23, 1000 frames	JPDA	0.905	0.316 m	0.742	51	14	0.972	0.952	71.09
RadarScenes seq. 25, 1000 frames	Proposed finite hold	0.938	0.160 m	0.929	6	12	1.000	0.942	2.88
RadarScenes seq. 25, 1000 frames	JPDA	0.938	0.153 m	0.929	6	12	1.000	0.942	46.61
RadarScenes seq. 28, 1000 frames	Proposed finite hold	0.878	0.184 m	0.670	60	52	1.000	0.895	6.63
RadarScenes seq. 28, 1000 frames	JPDA	0.726	0.418 m	0.690	147	201	0.949	0.812	111.45
RADIATE foggy 600 frames	Proposed finite hold	0.812	5.182 px	0.907	1	15	0.866	0.963	1.03
RADIATE foggy 600 frames	JPDA	0.751	10.354 px	0.688	27	25	0.890	0.932	8.03
nuScenes mini all scenes	Proposed finite hold	−1.026	1.595 m	0.095	487	123	0.184	0.260	38.58
nuScenes mini all scenes	JPDA	−1.147	1.605 m	0.092	478	126	0.169	0.261	434.66

**Table 6:** Host-side firmware-path modeled full-frame runtime under different target loads.

Tracks	Plots	Cold-Start Mean (ms)	Steady Mean (ms)	Steady Min (ms)	Steady Max (ms)	Frame-Period Usage (%)
0	0	9.190	9.189	9.189	9.190	18.4
1	4	9.465	9.580	9.510	9.683	19.2
2	8	9.774	9.793	9.575	9.905	19.6
4	16	10.430	10.521	10.046	10.746	21.0
6	24	11.148	11.631	10.922	11.998	23.3
8	32	11.935	12.590	11.916	12.900	25.2
10	40	12.698	13.468	12.570	13.937	26.9
12	48	13.560	14.896	13.587	15.837	29.8

## Data Availability

The authors’ full radar measurement data are not publicly released in the present version because the recordings are managed under laboratory data-management restrictions and contain unpublished project-specific measurement sequences. Representative raw I/Q frames used in the manuscript, the corresponding MATLAB R2025b/Python 3.12.11 processing scripts, parameter files, and generated result tables can be provided to the editor and reviewers for peer-review verification. The synthetic labeled range–Doppler detection benchmark is generated by the supplied script with a fixed random seed. Public benchmark scripts, parameter files, trajectory figures, and generated CSV tables are supplied for RadarScenes, the public RADIATE foggy sample, and nuScenes mini radar-only sequences, including proposed finite-hold and bounded-approximation JPDA tracking outputs. No public repository DOI is claimed for the authors’ measurement data in the present version.
